# Impact of Extra-Virgin Olive Oil Storage Conditions on Phenolic Content and Wound-Healing Properties

**DOI:** 10.3390/foods14122104

**Published:** 2025-06-15

**Authors:** Francesca Blasi, Maria Rachele Ceccarini, Stefano Bistarelli, Francesco Galli, Lina Cossignani, Desirée Bartolini, Federica Ianni

**Affiliations:** 1 Section of Food, Biochemical, Physiological and Nutritional Sciences, Department of Pharmaceutical Sciences, University of Perugia, 06126 Perugia, Italy; francesca.blasi@unipg.it (F.B.); lina.cossignani@unipg.it (L.C.); 2 Section of Morphological Biomolecular Nutraceutical and Health Sciences, Department of Pharmaceutical Sciences, University of Perugia, 06126 Perugia, Italy; mariarachele.ceccarini@unipg.it (M.R.C.); francesco.galli@unipg.it (F.G.); 3 Department of Mathematics and Computer Science, University of Perugia, 06123 Perugia, Italy; stefano.bistarelli@unipg.it

**Keywords:** EVOO storage, phenolic compounds, food analysis, wound-healing properties, tissue regeneration

## Abstract

Storage conditions significantly impact the quality and functional properties of extra-virgin olive oil (EVOO). This study investigated the impact of light and dark storage on the nutritional quality of Umbrian EVOO and its effectiveness in tissue repair. The research aimed to simulate real-world conditions occurring during transport, retail, and domestic storage. Light exposure accelerated EVOO oxidation, significantly affecting peroxide levels (ranging from 5.19 to 24.30 meq O_2_/kg of oil), total antioxidant capacity (measured spectrophotometrically, collectively ranging from 399.47 to 684.63 mg TE/kg of oil), and phenolic compound concentrations, particularly secoiridoids, lignans, and flavonoids (measured by HPLC, collectively ranging from 41.92 to 169.74 mg/kg of oil). Statistically significant differences (*p* < 0.01) were recorded between the control sample and the others in almost all cases, after storage. For instance, both light and dark exposure over a 24-month period resulted in a marked reduction (*p* < 0.01) in oleocanthal, pinoresinol, luteolin, and apigenin. Pigment levels were also affected, representing a rapid and cost-effective indicator of product oxidative degradation. The loss of phenolic compounds (especially oleacein and oleocanthal, which showed the most significant reductions of approximately 75% and 60%, respectively), impaired the EVOO’s wound-healing properties, affecting key tissue regeneration processes such as keratinocyte migration, hyaluronic acid synthesis, and angiogenesis. Notably, oleocanthal and oleacein, present at higher concentrations in fresh extracts, emerged as the primary contributors to the observed dermal effects and wound-healing processes, demonstrating a significant highest efficacy (*p* < 0.0001) in promoting wound closure. These findings underscore the critical role of EVOO storage in preserving its sensory properties and labile components with tissue repair and regeneration functions.

## 1. Introduction

Extra-virgin olive oil (EVOO) offers numerous health benefits due to its high content of monounsaturated fatty acids and bioactive components, especially phenolic compounds. This makes it a key component of the Mediterranean diet. The abundance of oleic acid (18:1 n-9), generally ranging from 55% to 83%, is the feature that distinguishes olive oil from other vegetable oils [1].

Even the minor components of olive oil (about 2% of the total oil weight) have received great attention from the scientific and industrial world. This fraction, generally indicated as the unsaponifiable fraction, includes various classes of chemical compounds, such as hydrocarbons, aliphatic and triterpenic alcohols, phytosterols, volatile compounds, tocopherols, carotenoids, pigments, and polyphenols [2]. The presence of antioxidant compounds in EVOO is noteworthy, as they play a significant role in enhancing oxidative stability and prolonging shelf life. The concentrations of these minor antioxidant components can vary considerably. For example, α-tocopherol can range from 10 to 250 mg/kg, total carotenoids can range from 0.5 to 31.5 mg/kg, and total phenolic compounds can range from 200 to 450 mg/kg. These variations depend on several factors. [3]. In general, bioactive compounds impact EVOO’s characteristics, including product stability and sensory and organoleptic properties. Furthermore, EVOO polyphenols have been increasingly studied for their nutrigenomics and functional properties [4], which may help to explain the oil’s health-promoting effects and its efficacy in preventing chronic and degenerative ailments, such as metabolic syndrome and diabetes, cardiovascular diseases, and cancer [5,6]. Additionally, these compounds are being explored as potential markers for determining geographical origin, cultivar, quality, adulteration, and stability during EVOO storage [7]. Tyrosol (Tyr; *p*-hydroxyphenylethanol, *p*-HPEA) and hydroxytyrosol (HT; 3,4-dihydroxyphenyl-ethanol, 3,4-DHPEA), along with their combination with other moieties to form secoiridoid derivatives, such as oleuropein and ligstroside, represent the main phenolic compounds. This phenolic fraction also includes oleacein (3,4-DHPEA-EDA) and oleocanthal (*p*-HPEA-EDA), followed by smaller amounts of lignans and flavonoids. The presence of these species is correlated not only with the high antioxidant and free-radical-scavenger properties but also with distinctive sensory attributes such as pungency and bitterness, key indicators of high EVOO quality.

While the concept of extra-virgin olive oil (EVOO) as a functional food is well understood, significant efforts are still needed to address the variations in production and storage processes. These variations impact both the chemical composition and sensory quality of the oil. For instance, exposure to light is known to promote the hydrolysis of triacylglycerols and accelerate the degradation of important compounds like phenols. In particular, oxidative and hydrolytic phenomena, as a consequence of oil exposure to light or heat, significantly worsen EVOO quality. This degradation is also accompanied by the loss of other minor constituents that contribute to its health benefits. For these reasons, to preserve oil shelf life and nutritional value, proper handling practices must be implemented across the supply chain and by consumers. While EU regulations set legal limits for parameters such as free acidity, peroxide value, extinction coefficients (K232 and K270), and fatty acid composition [8], no mandatory standards exist for polyphenol content. However, in May 2012, the European Food Safety Agency (EFSA) authorized the claim that “olive oil polyphenols contribute to the protection of blood lipids from oxidative stress” for oils containing no less than 5 mg of hydroxytyrosol (HT) and its derivatives (such as Tyr and oleuropein) per 20 g of olive oil. Nonetheless, a standardized approach to using polyphenol content as a quality marker during storage remains lacking, despite extensive research [9,10,11]. The natural variability of polyphenol content, the limited availability of certified reference standards, and the analytical complexity involved pose significant challenges to regulatory implementation.

In this context, the present study aimed to assess the quality parameters of an Umbrian EVOO, stored under light and dark conditions, to simulate supermarket or domestic environments over a timeframe of 24 months at ambient temperature. A comprehensive analysis was carried out to evaluate the analytical profile (in terms of acidity, peroxide value, fatty acid composition, and phenol profile), as well as the *in vitro* biological properties, measured in terms of total phenol content (TPC) and total antioxidant capacity (TAC). Additionally, the optimal storage duration and conditions were assessed as critical factors in preserving the color attributes of EVOO. Furthermore, the ability of phenolic extracts to promote wound healing in keratinocytes and endothelial cells was evaluated [12]. It is well established that alterations or disruptions in the healing process can lead to significant outcomes. Primarily, this includes the excessive formation of scar tissue, and at times, the chronicity of the wound [12]. Excessive scarring, such as keloids, may develop as a result of prolonged inflammation and the abnormal reorganization and remodeling of collagen within the extracellular matrix (ECM) [13].

On the other hand, chronic wounds are characterized by a prolonged inflammatory phase, elevated levels of ECM metalloproteinases, poor tissue oxygenation, increased bacterial load, and the reduced expression of growth factors [14]. These abnormalities in wound healing not only pose significant economic and social challenges but also severely impact the quality of life of affected individuals. As a result, there is a growing interest in developing innovative therapies to enhance and expedite wound healing. In this context, olive oil has been widely explored for its potential in wound therapy, as EVOO phenolic compounds show promise for wound treatment, both alone and in combination with other therapies [15]. This study is the first to investigate the efficacy of polyphenols on wound healing in both skin and endothelial tissues. The storage time under various real-world conditions was also studied. The findings underline the importance of proper oil storage in preserving its bioactive properties, which can maximize its therapeutic potential for skin regeneration and the repair of vascular damage.

## 2. Materials and Methods

### 2.1. Reagents and Oil Sample

All the employed solvents (hexane, methanol, acetonitrile, formic acid) were of analytical grade and purchased from Sigma Aldrich (Milan, Italy). Analytical standards of apigenin, vanillic acid, ferulic acid, *p*coumaric acid, veratric acid, and gallic acid were purchased from Merck Life Science (Milan, Italy), while 3-hydroxytyrosol, tyrosol, oleuropein, luteolin, kaempferol-3-O-glucoside, myricetin, and rutin were purchased from Extrasynthese (Genay, Lyon, France). Standards of oleacein, oleocanthal, and pinoresinol were purchased from PhytoLab (Vestenbergsgreuth, Germany). Folin–Ciocalteu reagent, 2,2′-azino-bis (3-ethylbenzothiazoline-6-sulphonic acid) diammonium salt (ABTS), (±)-6-hydroxy-2,5,7,8-tetramethylchromane-2-carboxylic acid (Trolox), 2,4,6-tris(2-pyridyl)-s-triazine (TPTZ), and 2,2-diphenyl-1-picrylhydrazyl (DPPH) were from Merck Life Science (Darmstadt, Germany). Water was purified using a Milli-Q Plus system from Millipore (Milford, MA, USA).

The reagents used for titration, including potassium iodide, glacial acetic acid (AcOH), chloroform, sodium thiosulfate, and starch, as well as the indicators phenolphthalein and starch, were purchased from Sigma-Aldrich (Milan, Italy).

Extra-virgin olive oil “Rocca di Casalina” was purchased from the Fondazione per l’Istruzione Agraria (University of Perugia) (Perugia, Umbria, Italy). It is a multivarietal EVOO obtained from different Umbrian cultivars (Moraiolo, Leccino, and Frantoio) harvested in November 2022.

The oil was delivered in a tin container and then aliquoted into clear glass bottles, sealed with metal screw caps, by minimizing the headspace volume. The oil in the tin container was immediately analyzed and used as the fresh, starting sample (t_0_). The aliquoted fractions were stored in the light (L) or dark (D) and subjected to analytical monitoring after three months (t_1-L_ and t_1-D_), eight months (t_2-L_ and t_2-D_), and twenty-four months (t_3-L_ and t_3-D_). These samples were compared to fresh oil (t_0_), which was analyzed immediately after opening the tin container. To simulate realistic indoor conditions (domestic storage, transport, and retail), the samples were exposed to natural light filtered through a window, taking into account variations in sunlight intensity throughout the day and across different seasons. Similarly, although the temperature was not continuously monitored, it is estimated to have ranged between 15 °C and 30 °C.

### 2.2. Colorimetric Analysis of EVOO Samples

The color of EVOO samples was evaluated with an EOPTIS CLM194 colorimeter (Metreo Solutions, Rome, Italy). The color difference function is based on the formula used in the CIE 1976 a, b color difference (CIELAB) ΔE*ab. For each entry, the CIELAB coordinates were displayed as follows: CIELAB ΔL* represents a lightness difference, CIELAB ΔC*ab represents a chroma difference, Δa* and Δb* components represent a difference between the measure and reference. In this color space, L* is the black/white coordinate, a* is the red/green color component, and b* is the yellow/blue coordinate. C* represents the derived magnitude, chroma, which is the distance from the lightness axis (L*) and starts at 0 in the center, and indicates the amount of saturation of a color. The software performs the color difference calculation and plots the CIE L*a*b* measure coordinates compared to the reference one (the fresh EVOO sample selected as the starting control, t_0_).

After measuring the samples of interest, the application software enabled the evaluation of color difference compared to the reference patch and classified the samples based on their resulting colors within user-defined acceptance limits.

### 2.3. Quality Index Determination

The free acidity and peroxide value were determined by titration, according to the official methods of European Commission Regulation [8].

Titratable acidity was expressed as oleic acid percentage (OA%), and the peroxide value was expressed as milliequivalents of active oxygen per kilogram of oil (mEq O_2_/kg).

### 2.4. Fatty Acid Determination by Gas Chromatography (GC)

The fatty acid (FA) composition of “Rocca di Casalina” oil was determined by high-resolution gas chromatography (HRGC) after the transformation of this fraction to the respective fatty acid methyl esters (FAMEs). In brief, 10 mg of oil was dissolved in hexane (2 mL), and then 2N methanolic KOH (0.5 mL) was added. After stirring, water was added, and the upper organic phase was dried over anhydrous sodium sulfate and then concentrated under a nitrogen stream.

A DANI GC1000 DPC gas chromatograph (Norwalk, CT, USA) equipped with a split/splitless injector and a flame ionization detector (FID) was used for FAME analysis [16]. The CP-Select CB for the FAME fused silica capillary column (50 m × 0.25 mm i.d., 0.25 μm f.t.; Varian, Superchrom, Milan, Italy) was used for the chromatographic separation. Data acquisition was performed by the Clarity software v. 3.0.06.589 (DataApex Ltd., Prague, Czech Republic). The following parameters were established: injector and detector temperature, 250 °C; oven temperature held at 180 °C for 6 min, then raised to 250 °C at 3 °C/min, and the final temperature was held for 10 min (total run time: 31 min). The carrier gas (He) flow rate was 1.0 mL/min; the injection volume was 0.4 µL with a split ratio of 1:70. Fatty acids were identified by comparing retention times with a standard solution containing 37 FAMEs. The amount of each FA was calculated as the area percentage value (Area%) (Supplementary Material).

The fatty acids considered in this study were palmitic (16:0), hypogeic (16:1 n-9), palmitoleic (16:1 n-7), margaric (17:0), margaroleic (17:1 n-9), stearic (18:0), oleic (18:1 n-9), Z-vaccenic (18:1 n-7), linoleic (18:2 n-6), linolenic (18:3 n-3) + arachidic (20:0), and gondoic (20:1 n-9) acids expressed as percentages of FAMEs.

### 2.5. Phenol Extraction

The extraction of phenols from EVOO was carried out according to a previously developed protocol [17]. In total, 10 g of oil was dissolved in 2.5 mL of hexane and then extracted by adding 5.0 mL of a water/methanol solution (60/40, *v/v*). The mixture was vortexed for 2 min and then centrifuged (Neya A 8-50, Remi, Mumbai, India) at 4000 rpm for 15 min. The two phases were separated, and the hexane phase was re-extracted by repeating the procedure. The hydroalcoholic phases were combined in the same tube and stored at 4 °C for 3 h to allow the decantation and separation of the residual oil from the mixture. Finally, the mixture was filtered under vacuum, and the obtained extract evaporated to dryness. The residue was dissolved in methanol to obtain a concentration of 1.0 mg/mL before spectrophotometric assays and chromatographic analysis.

### 2.6. Spectrophotometric Assays for the Determination of the Total Phenol Content (TPC) and Total Antioxidant Content (TAC)

Spectrophotometric assays were performed according to previous studies with slight modifications [18]. The Folin–Ciocalteu method was used for the TPC determination. The phenolic content of the EVOO extracts was determined, relying on a calibration curve of gallic acid. The absorbance was measured at 765 nm, and the results were expressed as mg of gallic acid equivalents per kilogram of oil (mg GAE/kg).

The TAC measurement was based on the determination of ferric reducing power (measured using FRAP assay) and free radical-scavenging activity (measured using DPPH- and ABTS-based assays).

To perform the FRAP assay, the extracts were incubated with the Fe3+-TPTZ complex for 30 min, and the absorbance was measured at 593 nm. The DPPH reagent was incubated with the phenol extracts for 30 min, after which the absorbance was measured at 517 nm. Finally, the ABTS reagent was mixed with the extracts and kept in the dark for 6 min, then the absorbance was measured at 734 nm.

The quantitative determination of the three assays was performed using a calibration curve of Trolox, and the results were expressed as mg of Trolox equivalent per kilogram of oil (mg TE/kg). All the UV spectra were recorded with a LAMBDATM UV–Vis spectrophotometer (PerkinElmer, Inc.; Waltham, MA, USA).

### 2.7. Polyphenol Characterization by High-Performance Liquid Chromatography–Diode Array Detector (HPLC-DAD)

The qualitative and quantitative profiles of phenols extracted from EVOO were determined by HPLC-DAD. The measurements were made on a Thermo Separation low-pressure quaternary gradient pump system coupled to a Spectra system UV 6000 LP Diode Array Detector (DAD) (Thermo Scientific, Waltham, MA, USA), supplied with a GT-154 vacuum degasser (Shimadzu, Kyoto, Japan), and a Rheodyne7725i injector (Rheodyne Inc., Rohnert Park, CA, USA) with a 20 µL stainless steel loop. Data acquisition was performed using the Xcalibur software (version 1.1, Thermo Finnigan Company, Chromatographic Specialties Inc., Brockville, CA, USA).

The following reversed-phase columns were screened to maximize the separation among the investigated polyphenols: Robusta C18 (250 × 4.6 mm i.d., 5 μm, 100 Å pore size, SepaChrom, Rho, Italy), Gemini C6-Phenyl (150 × 4.6 mm i.d., 5 μm, 100 Å pore size, Phenomenex, Milan, Italy), and Luna Omega PS C18 (250 × 4.6 mm i.d., 5 μm, 100 Å pore size, Phenomenex, Milan, Italy).

Each column was conditioned for 20 min before use with the selected mobile phase. The following optimized gradient program was applied: eluent A (water/AcOH-97.5/2.5 *v/v*) and eluent B (MeOH): 0 min—90% A; 28 min—60% A; 39 min—40% A; 50 min—10% A; 51 min—0% A. The flow rate was set at 1.0 mL/min, and the column temperature was set at 25 °C.

For the quantification of the analytes identified in the extracts, calibration curves were built using the corresponding standard solutions. To obtain the maximum sensitivity for each compound, the concentration was determined at the maximum wavelength of absorbance. Accordingly, a wavelength of 280 nm was used for HT, Tyr, *p-*coumaric acid, veratric acid, oleacein, oleuropein, and pinoresinol quantification, a wavelength of 250 nm was used for vanillic acid, while a wavelength of 340 nm was used for luteolin, kaempferol, and apigenin.

The calibration curves were characterized by a very high linearity as expressed by the R^2^ values (≥0.999). Details are reported in the following sections. A basic research validation of the established HPLC method was performed using two different control solutions of tyrosol and luteolin standards with nominal concentrations of 2.45 µg/mL and 0.5 µg/mL for tyrosol and concentrations of 2.45 µg/mL and 0.25 µg/mL for luteolin. The method was validated in terms of accuracy, precision, limit of detection (LOD), and limit of quantification (LOQ).

### 2.8. Cell Lines and Culture Conditions

Human immortalized keratinocyte cells (HaCaT, purchased from “Istituto Zooprofilattico Sperimentale della Lombardia e dell’Emilia Romagna”, I.Z.S.L.E.R.) were cultured in Dulbecco’s modified Eagle’s medium (DMEM; Euroclone), supplemented with 10% heat-inactivated fetal bovine serum (FBS, Invitrogen, CA, USA), 2 mM of L-glutamine (Sigma-Aldrich), and 1% penicillin/streptomycin (Sigma-Aldrich). Human umbilical vein endothelial cells (HUVECs; Lonza group, Switzerland).) were derived from three different donors and were seeded on plates coated with 0.1% porcine gelatin (Sigma-Aldrich). Cells were maintained in a 5% CO_2_ incubator at 37 °C and cultured up to passage 8 in Clonetics^®^ EGM^®^ Endothelial Growth Medium (Lonza group, Switzerland).

### 2.9. RT-qPCR Analysis

To evaluate the expression of genes related to skin moisture, HaCaT cells were seeded in a 6-well plate at a density of 5 × 10^4^ cells/cm^2^ and treated with different olive oil phenolic extracts, 0.5 µM in HT for 24 h. To evaluate the expression of genes related to angiogenesis, a concentration of 1 × 10^5^ cell/cm^2^ of HUVECs was used in a 6-well plate, and then all the different olive oil phenolic extracts, 0.5 µM in HT, were used to treat HUVECs for 24 h.

Total RNA was isolated using the RNeasy Mini Kit (Qiagen, Hilden, Germany) according to the manufacturer’s instructions. Once the RNA concentration and purity were confirmed, a total of 103 ng of total RNA was used for cDNA synthesis with a cDNA synthesis kit (Thermo Fisher Scientific, Waltham, MA, USA), according to the manufacturer’s instructions. The mRNA level was determined by qPCR with the SYBR Green reagent (Applied Biosystems, Thermo Fisher Scientific, Waltham, MA, USA). Expression values were normalized to those of GAPDH using the 2-ΔΔCt method.

The following PCR primers were used in this study:

HAS-1: Forward 5′-TGTATCCTGCATCAGCGGTC-3′, Reverse 5′-GCCGGTCATCCCCAAAAGTA-3′;
HAS-2: Forward 5′-GTGGATGACCTACGAAGCGA-3′, Reverse 5′-TACCCCGGTAGAAGAGCTGG-3′;
HAS-3: Forward 5′-TTGGCCTCATTCCTGTGTCC-3′, Reverse 5′-CTGGCAATAAGCTGTGTAGGC-3′;
Hyal-1: Forward 5′-TGTGGACGTGGATGTCAGTG3′, Reverse 5′-GTAGTAGGGGTAGGTGCCCA-3′;
Hyal-2: Forward 5′-ATGTGCAGAACTGGGAGAGC-3′, Reverse 5′-GGAAGCAAGTGTCTCGTCCA-3′;
Hyal-3: Forward 5′-TCTGGGCATCATAGCCAACC-3′; Reverse 5′-AGAGGCCGAGTTGGTTCTTG-3′;
VEGF: Forward 5′-GCAGAATCATCACGAAGTGGTG-3′, Reverse 5′-TCTCGATTGGATGGCAGTAGCT-3′;
TGF-β1: Forward, 5′-GCTCCACGGAGAAGAACTGCT-3′, Reverse 5′-CTGCTCCACCTTGGGCTTGC-3′;
FGF2: Forward 5′-AGAAGAGCGACCCTCACATCA-3′. Reverse 5′-CGGTTAGCACACACTCCTTTG-3′;
ANGPTL2: Forward 5′-GAACCGAGTGCATAAGCAGGA-3′, Reverse, 5′-GTGACCCGCGAGTTCATGTT-3′;
GAPDH: Forward 5′-ACAACTTTGGTATCGTGGAAGG-3′, Reverse 5′-GCCATCACGCCACAGTTTC-3′.

### 2.10. Wound-Healing Assay

The scratch test was performed using a CytoSelect^™^ wound-healing assay kit (Cell Biolabs, Inc., San Diego, CA, USA), making it possible to simulate a wound *in vitro*. This technique consists of performing a linear thin scratch “wound” (creating a gap) in a confluent cell monolayer to mimic, in a simple and inexpensive method, cell migration. In this study, the effect of phenolic oil extracts was tested simultaneously on HaCaT and HUVEC cell lines with different dilutions, 0.5 and 1 µM in HT for keratinocytes and 0.5 µM in HT for HUVECs.

Both cells were seeded in a 24-well plate at a final concentration of 3 × 10^5^ and incubated overnight. Inserts were removed after 24 h, leaving the wound field. After washing with PBS 1X to remove dead cells and debris [19], extracts, previously solubilized in specific complete medium for each cell line, were added to the cells. Complete medium alone was used to treat control cells (Ctr). After 18 h, the treatments were removed, and cells were washed twice with PBS 1X. Following this, 300 µL of the fixing solution was added to each well and left for 10 min. The fixing solution was then removed, and each well was washed twice with PBS. Then, 500 µL of cell stain solution was added to each well to stain the cells. After 15 min, the cell stain solution was removed, and each well was washed twice with 500 µL of PBS 1X and deionized water.

The wound area was calculated by manually tracing the cell-free area in captured images using the public domain software ImageJ (bundled with Java 8, NIH, Bethesda, MD, USA) [20]. The closure increases as cells migrate over time. To measure the % closure, the migration cell surface area was determined for each experiment (migration cell surface = total surface area − cell-free area). The percentage closure of the wound field was calculated using Equation (1):

(1) % closure = (total surface area − cell-free area)/total surface area × 100

where total surface area means the area immediately after removing the insert, and cell-free area means the white area in the photograph after 18 h of growing. Migration into the wound field was determined as previously described [21], and representative images of the control and treated cells were taken after 18 h. Three independent experiments were performed in duplicate. Three fields per image were analyzed in duplicate, and the analysis was performed in a blinded manner.

### 2.11. Statistical Analysis

The results are expressed as mean value ± standard deviation (n = 3). Statistical significance was measured through one-way analysis of variance (ANOVA), followed by Tukey’s honestly significant difference *post hoc*. Graph Pad PRISM package 9.2.0.332 (GraphPad Software for Science, Inc., San Diego, CA, USA) was used as the statistical software. Values with *p* < 0.01 were considered significant. Multivariate statistical analysis, used to discriminate significant differences between groups, was performed by MetaboAnalyst (v 6.0). All data were normalized to the median and Pareto-scaled prior to further analysis.

Chemometric analysis was obtained using the MetaboAnalyst 6.0 web platform on the normalized dataset. Pareto scaling based on the square root of the standard deviation was applied as the scaling factor, providing an intermediate scaling effect between the no scaling and unit variance scaling. Principal Component Analysis (PCA) was applied to follow the distribution features of the dataset. Partial Least Squares–Discriminant Analysis (PLS-DA) was used to determine the variable importance in projection (VIP) of each analyte. Only compounds with a VIP value > 1.0 were considered significantly different between the investigated groups.

## 3. Results

### 3.1. Colorimetric Analyses of EVOO Under Storage

The impact of storage conditions on the color properties of EVOO was assessed through colorimetric analysis in the CIELAB color space. Color determination is significant for consumer perception, even though it is not included in standard quality parameters. The primary pigments that give EVOO its green and yellow hues are chlorophylls and carotenoids, respectively. Additionally, these compounds are important for the stability of the oil. The effect of the investigated EVOO storage on the color properties was determined on unfiltered samples by colorimetric analysis performed in the CIELAB space. Color determination plays an important role in consumer perception, even though it is not included in quality standard parameters. The primary pigments, responsible for the green and yellow color in extra-virgin olive oil (EVOO), are chlorophylls and carotenoids, respectively. Furthermore, these compounds play a crucial role in oil stability.

Sample t_0_ was used as the control. The data indicated a low dispersion for the three investigated parameters: L* (black/white coordinate), a* (red/green color component), and b* (yellow/blue coordinate). All samples were located in the zone of the yellow-greenish color. However, even if the L* value fell within a strict interval of variation (85.39–87.44), indicating a generally fairly bright color, a slight increase could be observed, more pronounced for light-exposed samples. This result, consistent with the existing literature, suggests a gradual lightening of the oil, likely due to oxidative and pigment degradation [22]. Accordingly, the intense green color of chlorophyll and the orange hue of carotenoids tend to fade upon light exposure, giving the sample a lighter appearance. In contrast, dark-stored samples showed slightly lower L* values, confirming that light exposure contributes more significantly to the brightening effect.

Concerning the a* value, all samples showed negative values, indicating a tendency toward green with minimal variation across the samples. Over time, the absolute values of a* slightly increased, especially in dark-stored samples. Although this change was minimal, it suggests that the chlorophyll degradation process was slower in dark conditions than in samples exposed to light. A significant decline in b* values was observed in light-exposed samples, compared to the control sample. The strong yellowish tone of the fresh EVOO sample (b* = 72.1) was lost over time, with a convergence towards paler yellow, more pronounced for samples exposed to light. This could be attributed to the degradation of carotenoids throughout storage as previously described by Betriu and co-workers [23]. Several factors could be called into play to explain the observed variations. Chlorophylls, for instance, act as photosensitizers and can generate singlet oxygen, which accelerates oxidation and promotes oil deterioration. Furthermore, when exposed to light, chlorophyll undergoes chemical changes that result in a natural degradation process called pheophytinization. During this process, the central magnesium ion (Mg^2+^) in the porphyrin ring is replaced. This transformation plays a significant role in the loss of the original color of extra-virgin olive oil (EVOO). Similarly, light exposure can accelerate the oxidative degradation of carotenoids, resulting in the formation of colorless or less intensely colored compounds, thereby affecting the colorimetric parameters.

Finally, the dE*ab parameter, which quantifies the overall color difference from the reference EVOO (t_0_), showed a significant increase over time, particularly evident for prolonged light exposure. T_2-L_ and t_3-L_ showed the most noticeable color shifts. The color properties of the investigated samples are summarized in Table 1. Significantly high statistical differences (*p* < 0.01) were recorded in almost all cases.

### 3.2. Quality Parameters and Fatty Acid Composition

The quality parameters of the samples stored at room temperature were assessed based on free acidity and peroxide value, following the official methods outlined in the Regulations of the European Commission [8]. These two parameters are commonly used to categorize olive oil.

In all the examined conditions, the free acidity values were below the maximum limit established for EVOO, which is 0.8% (Table 2). A slight decrease in acidity was observed in samples t_1_ and t_2_ compared to t_0_. This finding contrasts with observations from other authors, who generally observed an increase in acidity value as the storage time increased. Gargouri and co-workers stated a small increase in free acidity from 75 to 180 days of Chemlali EVOO according to the storage time [24]. Similarly, Pristouri and co-workers [25] also observed a slight increase in free acidity after 12 months of storage.

A reduction in the free acidity value could be attributed to the peculiar storage mode of the oil, which can contribute to the stabilization or even slight reduction in acidity compared to the control, particularly during the initial months of storage, as also reported by Lazarou [26]. Moreover, the progressive degradation of free unsaturated fatty acids to form oxidation products could be plausibly hypothesized.

The analysis revealed no statistically significant differences among the three types of samples (t_0_-t_2_), indicating that free acidity was not affected by storage duration or exposure. All the samples were classified as extra-virgin olive oils according to established reference standards. In addition, these values agree with those recently reported by Esposto and co-workers [10], ranging from 0.25 ± 0.01 to 0.44 ± 0.02, determined on a set of 20 EVOO samples at their initial time determination. In agreement with Pristouri [25], our result clearly indicates that free acidity alone cannot be used as a good indicator to establish the quality of olive oil.

The physicochemical changes associated with oxidation processes were monitored by the measurement of the peroxide value. A very low peroxide value is desirable, as this parameter, along with free acidity, is conventionally used as an indicator of the ongoing oxidation and, therefore, as a potential signal of the start of oil rancidity. Regarding oxygen, EVOO must have a peroxide index ≤ 20 mEq O_2_/kg [8]. Lower values are generally indicative of higher quality and better stability.

Storage conditions play a critical role in the oxidative stability of EVOO, as degradation can occur in the dark through autoxidation and can be accelerated by light via photo-oxidation. Our evaluation revealed a significant increase in the peroxide value of samples stored in glass containers exposed to light for three months (t_1-L_) compared to fresh EVOO (t_0_). However, this deterioration was not observed to the same extent in samples stored in the dark (t_1-D_) (Table 2). These findings indicate that photo-oxidation is a major contributor to peroxide value increase, exceeding the effects of autoxidation. This is likely due to the presence of dissolved oxygen in the oil, which drives oxidation at a slower rate in the absence of light, combined with chlorophyll pigments that act as photosensitizers, accelerating oxidation under light exposure.

Our results align with previous studies, which consistently show greater losses in olive oil quality when stored in light-exposed conditions compared to storage in darkness [25,27].

A remarkable deterioration in the oxidation state was observed in oil samples stored in the dark (t_2-D_) after eight months (Table 2). This suggested that non-photo-assisted oxidative processes became the dominant mechanism over photo-assisted ones. In contrast, no substantial changes were detected in the sample exposed to light (t_2-L_), indicating that oxidative stability had been achieved. This trend is more clearly illustrated in Figure S1 (Supplementary Material). Although an increase in oxidation was observed under both light and dark storage conditions. However, all samples still fell within the classification of “extra-virgin olive oil” according to European standards. The situation changed drastically for the t_3_ samples, indicating a severe deterioration in EVOO quality and integrity (Table 2). Surprisingly enough, the degradation was even more pronounced in the dark-stored sample, suggesting again that prolonged storage seems to favor non-photo-assisted oxidative processes over time. This unexpected result could be attributed to the initial antioxidant effect of chlorophyll, which is well-known to protect oils from autoxidation when stored in the dark, as demonstrated in previous studies [28]. It is reasonable to hypothesize that over time, there may be a depletion of chlorophyll and other antioxidant components [29], leading to a loss of antioxidant potential and ultimately affecting the stability of the oil samples during extended storage.

Tukey’s multiple test for free acidity (%) revealed statistically significant differences (*p* < 0.01) between t_0_ and all other samples, as well as between t_3_ and all other time points. However, a closer similarity was observed between t_0_ vs. t_1-D_ and t_0_ vs. t_2-D_, with significant differences at *p* < 0.05. Concerning the peroxide value, statistically significant differences (*p* < 0.01) were generally observed across the samples. The only exception was between t_1-L_ and t_1-D_, where no significant difference was found. Additionally, a significant difference (*p* < 0.05) was observed between t_2-L_ and t_2-D_.

This study emphasizes how crucial storage conditions are for maintaining the quality and stability of EVOO. Although storage in the dark is typically expected to limit oxidation, the observed oxidation, ascribable to oxygen ingress through metal screw caps that are not fully airtight, as often occurs in domestic environments, suggests that oxygen exposure remains a significant factor contributing to oil deterioration. Accordingly, the use of dark glass bottles with airtight sealing as well as proper storage conditions could minimize side reactions. Finally, recommending a specific shelf life after opening the container would allow consumers to utilize a high-quality product.

The fatty acid composition was determined by gas chromatography (GC) as fatty acid methyl esters (FAMEs). The composition reported in Table S1 (Supplementary Material) for samples t_0_, t_2-L,_ and t_2-D_ indicated that the main fatty acids in “Rocca di Casalina” oil include palmitic acid, oleic acid, and linoleic acid. Moreover, the GC analysis revealed the presence of the *trans*-vaccenic acid, which has recently been the subject of heated controversy for its potential health benefits [30,31].

According to the EU regulations, fatty acid composition fell within the intervals required. This result was also confirmed for samples stored in both light and darkness after three and eight months. Only slight changes in fatty acid composition were observed, in line with data reported in the literature [24].

### 3.3. Evaluation of the Polyphenol Content by Spectrophotometry

The concentration of total polyphenols (TPC) and the antioxidant capacity (TAC) of EVOO have been traditionally evaluated via spectrophotometry [32]. Depending upon the mechanism involved, the antioxidant assays are generally classified as Hydrogen Atom Transfer (HAT)- or Single-Electron Transfer (SET)-based assays. To have a more realistic assessment, the antioxidant capacity of complex polyphenol mixtures, such as EVOO, is generally determined using a combination of a few assays involving different chemical reactions [33]. For this reason, in the present study, the radical-scavenging capacity was measured through mixed mode (HAT/SET) assays, ABTS and DPPH, and the non-radical SET-based method, FRAP: the information obtained by integrating the different mechanisms could support a more meaningful evaluation of the potential beneficial health effects of the extracts. As shown in Table 3, the TPC value was the highest for the t_0_ sample, followed by an actual decrease after three months of storage and a slight increase after eight months of storage. This trend was generally shared by all the related antioxidant assays, with the sole exception of ABTS, which gave a relatively steady response. The partial degradation of secoiridoid and other phenol derivatives over time may contribute to the formation of intermediate or final products, which could, in turn, influence the specific mechanisms underlying each assay. A drastic reduction in the TPC and antioxidant activity was instead found for the t_3-L_ and t_3-D_ samples, indicating an extensive degradation of phenolic compounds over time, likely due to oxidative and hydrolytic processes. After 24 months of storage, these transformations may lead to the formation of inactive or less bioavailable derivatives, resulting in a substantial loss of antioxidant potential. Prolonged exposure to light reduces the phenolic content and antioxidant capacity of EVOO, as evident in sample t_3-L_.

Tukey’s multiple test indicated that the results for t_0_ were significantly higher than all subsequent time points, measured by TPC and DPPH. Notably, both the t_1-L_ and t_1-D_ samples showed a substantial reduction in values, making these samples indistinguishable from one another across the various assays. Despite the slight increase observed for t_2-L_ and t_2-D_ compared to t_1_ for both TPC and DPPH, the values remained significantly lower than those at t_0_. In line with earlier observations, no differences were detected in the ABTS assay between samples stored for three or eight months in light or in the dark.

The comparison of all possible pairs of means highlighted differences between t_0_, t_1-L,_ t_1-D,_ and t_2-L_. No differences between t_0_ and t_2-D_ were found, even at a higher threshold (*p* < 0.05). A similar observation could be made in the evaluation of the FRAP assay, where similar trends (*p* > 0.05) were observed between the samples monitored in the same period (that is, t_1-L_ vs. t_1-D_ and t_2-L_ vs. t_2-D_).

In all spectrophotometric assays, samples t_3-L_ and t_3-D_ showed the lowest values, with t_3-L_ being significantly different from all other time points, and t_3-D_ being slightly higher but significantly different from t_0_. This highlights the severe oxidative degradation observed.

The correlation plots (Figure S2, Supplementary Material) showed a strong relationship (R^2^ > 0.80) between TPC and both DPPH and FRAP, whereas a weaker correlation was observed with the ABTS assay (Figure S2A). The lower reliability of ABTS also affected its correlation with DPPH (R^2^ _DPPH vs. ABTS_ = 0.3851), while higher correlations were found between the remaining pairs (R^2^ _DPPH vs. FRAP_ = 0.8592; R^2^ _ABTS vs. FRAP_ = 0.6339) (Figure S2B). Overall, the observed trends suggest that oil samples exposed to both light and dark underwent similar changes in phenol content and antioxidant properties over time. This is further supported by the strong correlation (R^2^ = 0.778) between the FC assay and TAC (calculated as the sum of the DPPH, ABTS, and FRAP assays; Figure S2C). The TPC and TAC values showed moderate (R^2^_TPC vs. HPLC_ = 0.5975) and low (R^2^_TAC vs. HPLC_ = 0.2299) correlation values with the polyphenol concentrations determined by HPLC-DAD. Remarkably, after excluding sample t_3_ from the dataset, these correlations improved significantly, with R^2^_TPC vs. HPLC_ = 0.821 and R^2^_TAC vs. HPLC_ = 0.5184 (Figure S2D). This observation highlights the significant impact that storage time has on the deterioration of oil quality. In the early stages of storage, the decrease in phenolic content closely paralleled the decline in antioxidant activity. However, as the storage time increased, the formation of secondary products could complicate the interpretation of this trend due to interferences with the assays and the potential contribution to the final outcomes.

Even more interesting is the correlation between specific analytes and various antioxidant assays. In particular, most of the investigated analytes, especially secoiridoid derivatives, lignans, and flavonoids, showed a high correlation with the DPPH assay (R^2^ > 0.78). A moderate correlation (R^2^ > 0.6) with ABTS and a low correlation (R^2^ < 0.45) for FRAP were found. These findings suggest that the HAT mechanism would prevail over the SET one. The results indicate that the catechol group and the position of hydroxyl functions in the molecules may affect their ability to scavenge radicals by the donation of hydrogen atoms to free radicals. Conversely, low correlation coefficients (R^2^ < 0.35) were consistently observed between the concentrations of tyrosol, hydroxytyrosol, and phenolic acids as measured by HPLC-DAD and the outcomes of the spectrophotometric assays.

This may be attributed to the inherently lower antioxidant activity of hydroxybenzoic acid derivatives, compared to other classes of phenols [34], and the effective concentration ranges required for these compounds to exhibit measurable activity [33].

The results indicate that the qualitative and quantitative profiles of phenols in olive oil have a more significant impact on its oxidative stability than the total phenolic content alone. This is crucial when determining the recommended daily intake for preserving the oil’s stability and nutritional value. Phenolic compounds also contribute significantly to key organoleptic properties, such as pungency and bitterness.

Finally, this study demonstrated that the prolonged and improper storage of EVOO, whether under light exposure or in darkness, induces irreversible changes due to the degradation or loss of polyphenols and secoiridoid derivatives. Such alterations can adversely affect the oil’s composition within the recommended period of consumption (“best before”) of 12 or 18 months.

### 3.4. Storage Conditions Modify the Polyphenol Composition of EVOO Samples

EVOO samples, under different storage conditions, were characterized by their potential health benefits according to the phenolic content. The primary focus of this study was to evaluate the changes in the most common polyphenols in EVOO during storage. To achieve this, qualitative and quantitative HPLC analyses were carried out. Furthermore, since polyphenols are known for their potential role in oxidative processes, *in vitro* spectrophotometric analyses were performed to investigate the oxidative stability that these bioactives provide to the oil throughout its storage.

Starting from a recently developed reversed-phase HPLC-DAD method [17], the separation of the most characterizing EVOO polyphenols [35], such as oleuropein, HT, luteolin, and apigenin, to name a few, was optimized. In particular, we aimed to maximize the separation between the investigated species while achieving the full chemoselectivity missing in our previous work for the pair kaempferol/apigenin [17]. The use of conventional C18 columns for the qualitative and quantitative analyses of EVOO has been extensively reported in the literature [9,36,37], while to the best of our knowledge, there are no specific applications with the use of hexyl–phenyl-based stationary phases.

**Figure 1:**
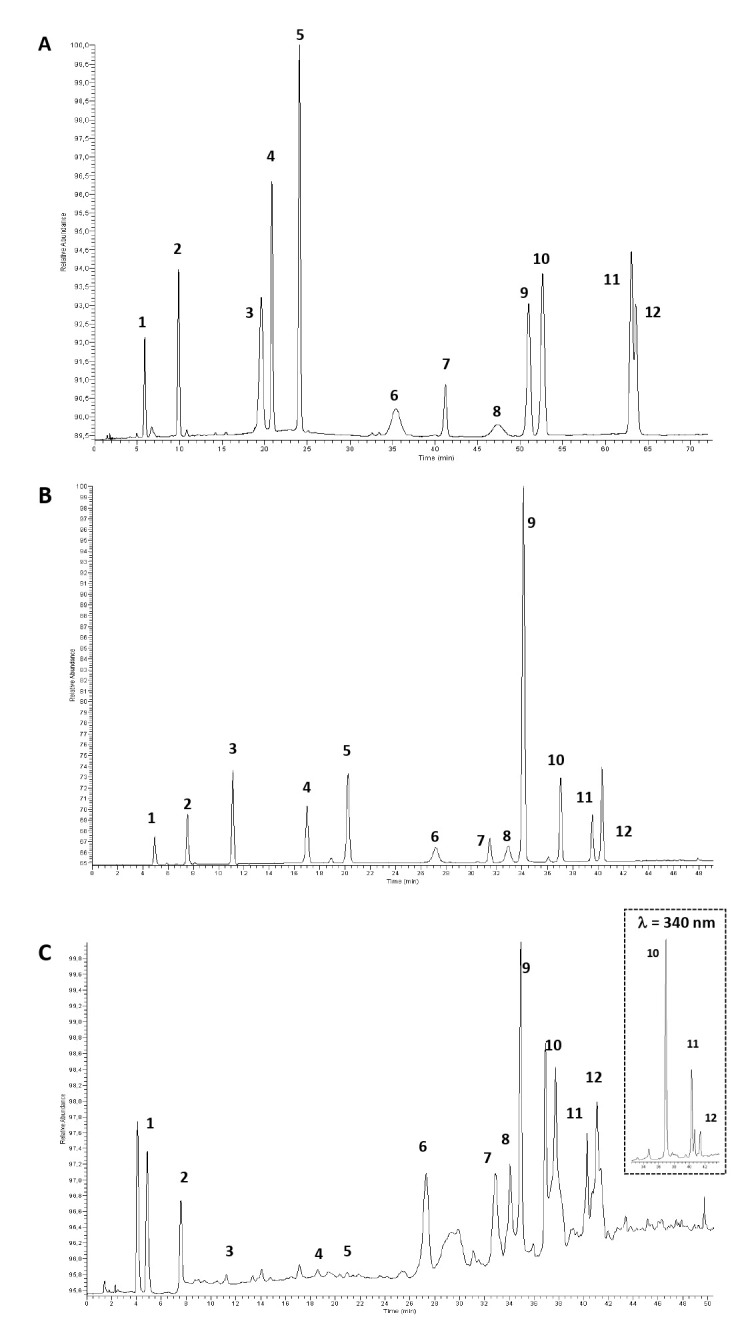
Chromatographic profile of a standard mixture of polyphenols with the Gemini C6-Phenyl column in (**A**) starting conditions (gradient: eluent A = water/AcOH, 97.5:2.5 *v/v* and eluent B = MeOH/ACN, 50:50 *v/v*: 0–5 min, 5% B, 5–30 min, 20% B; 30–35 min, 25% B; 35–70 min, 65% B; 70–75 min, 100% B); (**B**) optimized conditions (gradient: eluent A = water/AcOH, 97.5:2.5 *v/v* and eluent B = MeOH: 0 min, 10% B, 0–28 min, 40% B; 28–39 min, 60% B; 39–50 min, 90% B); (**C**) exemplary chromatographic profile of the phenolic extract from the t_0_ EVOO sample. Peak #: 1 = hydroxytyrosol, 2 = tyrosol, 3 = vanillic acid, 4 = *p-*coumaric acid, 5 = veratric acid, 6 = oleacein, 7 = pinoresinol, 8 = oleocanthal, 9 = oleuropein, 10 = luteolin, 11 = apigenin, and 12 = kaempferol. The inset represents the profile of luteolin, kaempferol, and apigenin at a wavelength of 340 nm.

To this end, three columns were screened (see details in Section 2.7 ) with a starting elution gradient. Although baseline separation between apigenin and kaempferol was not achieved under these preliminary conditions, the Gemini C6-Phenyl proved to be the best compromise in terms of chemoselectivity (Figure 1A). This performance was likely due to the phenyl groups in the stationary phase, which trigger selective π–π interactions with the aromatic rings of the polyphenols. Furthermore, hydrophobic interactions involving the hexyl linker directly attached to the silica surface play a significant role in overall retention. The gradient profile was then carefully fine-tuned to enhance the separation of the target analytes, with a particular focus on the most critical pair of peaks. The optimal condition significantly improved system chemoselectivity, while also reducing the elution time (Figure 1B). The HPLC-DAD analysis of the polyphenol extracts from EVOO subjected to different storage times and conditions evidenced important variations in the contents of the investigated compounds: phenolic alcohols (hydroxytyrosol and tyrosol) and secoiridoids derivatives (oleuropein, oleoacein, oleocanthal), phenolic acids (vanillic acid, *p*-coumaric acid, veratric acid), flavonoids (luteolin, apigenin, kaempferol), and lignans (pinoresinol).

**Figure 2:**
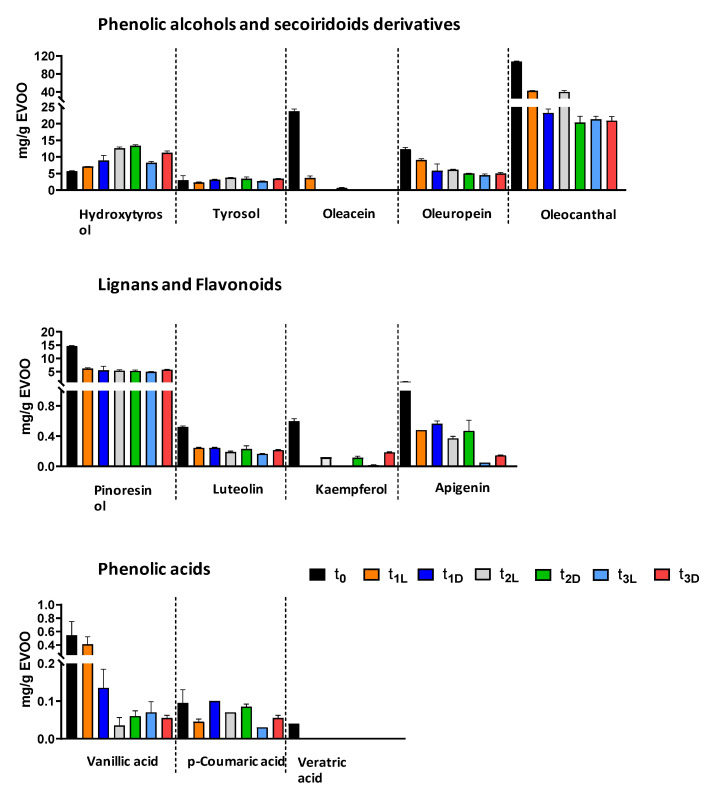
Trend of the major polyphenol classes in “Rocca di Casalina” EVOO before storage (t_0_) and after three, eight, and twenty-four months of storage at room temperature, both in light (t_1-L_, t_2-L,_ and t_3-L_) and in darkness (t_1-D_, t_2-D,_ and t_3-D_).

The results showed an overall decrease in the initial concentration of the major analyte classes. A significant reduction was observed for oleocanthal, pinoresinol, luteolin, and apigenin compared to t_0_. They exhibited a sharper decline in the samples stored in the dark for three months (t_1-D_) compared to those kept in the light for the same period (t_1-L_) (Table 4, Figure 2). Oleacein was completely degraded in the dark-stored samples, whereas kaempferol was detectable only in t_1-D_, not in the t_1-L_ extract. This decreasing trend continued after 8 and 24 months, with varying degrees of reduction in compound concentrations. The decline was particularly pronounced for oleacein and vanillic acid in both light- and dark-stored samples. Additionally, *p*-coumaric acid showed a gradual decrease over time, while the initially low concentration of veratric acid was completely lost. Statistical analysis (Table 4) revealed significant variations in polyphenol content at t_0_ compared to later time points, with highly significant differences in some cases (*p* < 0.01).

Among the secoiridoid derivatives, oleacein and oleocanthal experienced the most significant losses, with reductions of 75% and 60%, respectively, after just three months of storage. In contrast, oleuropein proved to be the most stable compound in this group, showing only a 25% decrease. The pronounced degradation of oleacein and oleocanthal can be attributed to their structural characteristics, specifically, the presence of two aldehyde functions in an open configuration. It likely increases their reactivity and susceptibility to degradation.

Overall, the observed decline in phenol levels aligns with the findings reported in the literature. Mousavi et al. [38] reported a slight reduction in the content of flavonoids and lignan phenols in EVOO subjected to various storage conditions over time. A more pronounced decrease was observed for secoiridoid derivatives, suggesting their more active involvement in the oxidative processes. Castillo-Luna and co-workers [39] highlighted how the reduction in oleuropein, oleacein, and oleocanthal, after 12 months of storage in darkness at 20 °C, was strongly dependent on the initial phenolic profile. In particular, EVOO with high concentrations of oleuropein and other compounds, such as ligstroside aglycone isomers, exhibited greater stability compared to those with lower phenolic content. Conversely, EVOO stability decreased as the content of oleacein and oleocanthal increased [40].

The two phenolic alcohols, HT and tyrosol (Tyr), deserve a separate discussion. The concentrations of these compounds exhibited a slight increase during storage, concomitant with the decline in secoiridoid derivatives, as shown in Table 4 and Figure 2. Although the initial levels of HT and Tyr were generally low and remained so throughout both light and dark storage conditions, their increasing concentrations suggest ongoing hydrolytic processes involving the primary secoiridoids, whose relative content diminished under all storage conditions. It is widely acknowledged that the content of HT and tyrosol rises as EVOO ages [39,41]. However, it is important to note that the conversion of secoiridoid derivatives to both respective alcohols did not follow a proportional relationship, implying that other degradation processes may occur over time.

Method validation was performed to assess the adequacy of the HPLC analysis protocol. Research-level investigation yielded satisfactory and consistent results, meeting the criteria required to confirm its suitability for the intended purpose (Tables S2 and S3, Supplementary Material).

**Figure 3:**
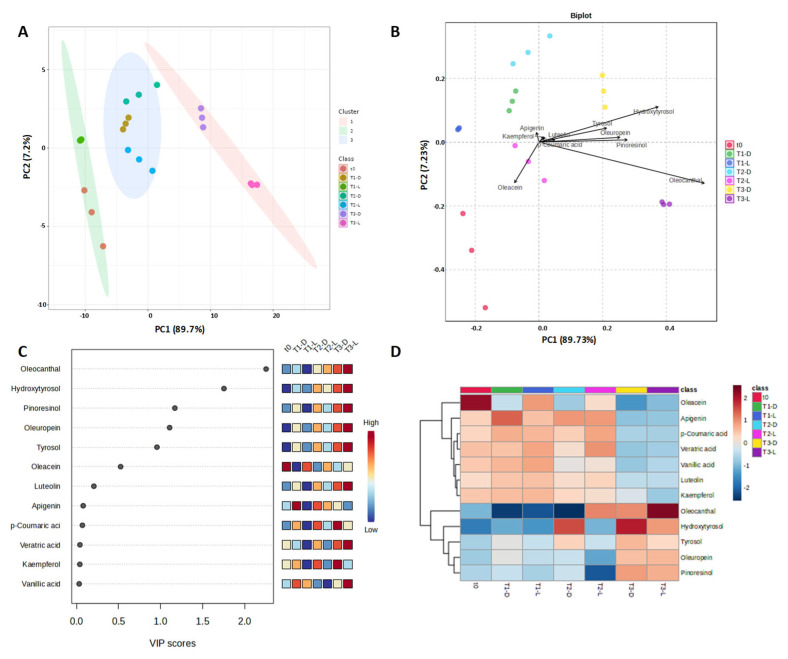
Chemometric analysis on normalized data. (**A**) PCA score plot with clusters; (**B**) corresponding biplot with longer vector indicating greater influence on the principal components; (**C**) variable importance in projection (VIP) scores, emphasizing the top features with the most significant contributions to the model’s performance; (**D**) heat map displaying, in red, the most significant features (in rows) for each individual sample (in columns).

The analysis of the PCA score plot revealed a distinct distribution of EVOO samples, linked to their storage conditions. The first principal component (PC1) accounted for 89.73% of the variability, highlighting three primary clusters ( Figure 3 A). It identified similar phenol composition in the t_0_ and t_1-L_ samples, which differed from t_1-D_, t_2-L,_ and t_2-D,_ and even more from the t_3-D_ and t_3-L_ samples. Overall, PC1 describes the main variation due to the EVOO aging, while the PC2 axis appears more sensitive to subtle differences arising from different storage conditions (light vs. darkness).

The scatterplot obtained by merging the score and loading plots (Figure 3B) provides a comprehensive representation of the critical variables for EVOO differentiation. Oleacein and oleocanthal vectorial annotations point in opposite directions to other compounds. This suggests that these molecules vary differently from other phenols. Their decrease over time and the increase in HT and Tyr are consistent with secoiridoid degradation processes. Notably, oleacein is significant for the t_2-L_ and t_0_ samples, while oleocanthal is the major discriminant for t_3-L_. The same vector orientation of HT, Tyr, oleuropein, and pinoresinol along PC1 indicates that their variations are closely related, especially in samples stored in the dark. The proximity of apigenin, kaempferol, and luteolin to the origin of the axis indicates, instead, their relatively minor role in sample separation. To further explore the relationship between treatments and significant metabolites, a Partial Least Squares–Discriminant Analysis (PLS-DA) regression was conducted to determine the variable importance in projection (VIP). The VIP plot generated individual analytes ranked based on their power to discriminate samples over time (Figure 3C). The higher the VIP scores of the analytes, the more important their contribution to establishing differences between groups. The results of the VIP analysis revealed that oleocanthal, hydroxytyrosol, pinoresinol, and oleuropein were particularly significant and differentiating the samples, whereas Tyr and oleacein gave a lower contribution to the model.

The hierarchical clustering in the heatmap images in Figure 3D confirms the decrease in the concentrations in the majority of the phenolics during sample storage. Interestingly, oleocanthal emerged as the most relevant polyphenol among those identified in sample t_3-L,_ while oleacein proved to be a key discriminant for the t_0_ samples, with a substantial reduction in its levels during storage under both dark and light exposure conditions.

### 3.5. Influence of Oil Phenolic Extracts on Factors Impacting Skin Moisture

**Figure 4:**
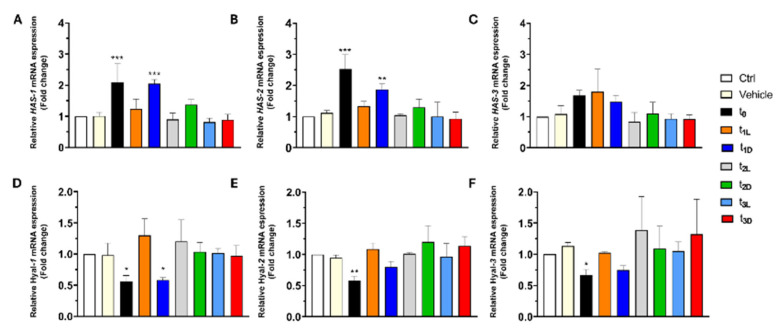
Effects of oil phenolic extracts on parameters that can affect skin moisture in HaCaT cells. (**A**–**F**) The mRNA levels of HAS-1, HAS-2, HAS-3, Hyal-1, Hyal-2, and Hyal-3 in 24 h phenolic extract (0.5 µM of HT)-treated HaCaT cells were determined by quantitative real-time PCR. The fold change represents the ratio of the increased mRNA expression levels of the treated groups to the mRNA expression level of the control group (Ctrl). All results are expressed as the mean ± standard deviation. One-way ANOVA: *** *p* < 0.0001; ** *p* < 0.01; * *p* < 0.05 compared with the Ctrl.

The effects of phenolic extracts from EVOO on key factors associated with skin moisturization were evaluated. HaCaT cells, which produce hyaluronic acid (HA), were used as a model to assess the skin moisturizing effects of different phenolic extracts. To determine the effects of these extracts on the expression of mRNA related to skin moisture, we assessed the mRNA expression of three hyaluronan synthase genes (HAS-1, -2, and -3) after treatment with 0.5 in HT phenolic extracts for 24 h. The mRNA level of HAS-1 and -2 increased following treatment with the t_0_ and t_1-D_ extracts with a very high significance level, whereas HAS-3 expression did not significantly increase after all the treatments (Figure 4A–C).

On the other hand, to investigate cell proliferation, migration, and differentiation, we considered the mRNA expression of hyaluronidase (Hyal) genes, which are involved in HA degradation in the ECM. Hyal-1, -2, and -3 expression was significantly reduced by t_0_ treatment (Figure 4D–F), and only Hyal-1 decreased after treatment with t_1-D_, while other treatments had no significant effect on Hyal gene expression (Figure 4D–F). Put simply, keratinocytes treated with t_0_ and t_1-D_ exhibited an increased expression of two enzymes responsible for HA synthesis (HAS-1 and -2) and a decreased expression of an enzyme (Hyal-1) involved in HA degradation, suggesting a potential skin-moisturizing effect of these EVOO phenolic extracts, as they help maintain HA levels, a key molecule for skin hydration and elasticity. These results confirm that the phenolic compounds contained in olive oil are natural and sustainable ingredients for the skin’s anti-aging effect and that the storage and monitoring of these phenolic ingredients is of paramount importance to ensure the efficacy and safety of cosmetic products.

### 3.6. Angiogenesis Promotion by Oil Phenolic Extracts in HUVECs

**Figure 5:**
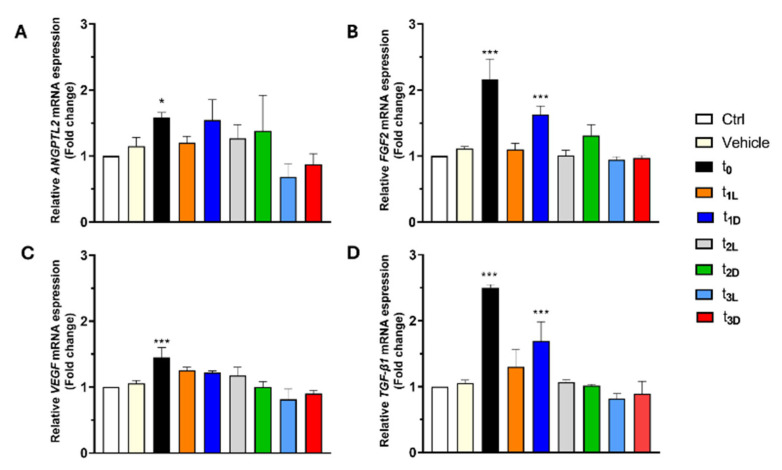
Effects of oil phenolic extracts on angiogenesis in HUVECs. The mRNA levels of ANGPTL2 (**A**), FGF2 (**B**), VEGF (**C**), and TGF-β1 (**D**) in 24 h phenolic oil extract (0.5 µM of HT)-treated HUVECs were determined by quantitative real-time PCR. The fold change represents the ratio of the increased mRNA expression level of the treated groups to the mRNA expression level of the control group (Ctrl). All results are expressed as the mean ± SD. One-way ANOVA: *** *p* < 0.0001 and * *p* < 0.05 compared with the Ctrl.

The effects of phenolic extracts from EVOO on HUVEC angiogenesis were also investigated. The results of RT-qPCR analysis showed that only Angiopoietin-like protein 2 (*ANGPTL2*) and vascular endothelial growth factor (*VEGF*) mRNA expression increased after 24 h of t_0_ treatment (Figure 5A,C). Additionally, the mRNA expression of fibroblast growth factor 2 (*FGF2*) and transforming growth factor beta (*TGF-β1*) was upregulated after 24 h following both t_0_ and t_1-D_ treatments (Figure 5B,D).

### 3.7. Oil Phenolic Extracts Stimulate Wound-Healing Processes in Keratinocytes and Endothelial Cells

**Figure 6:**
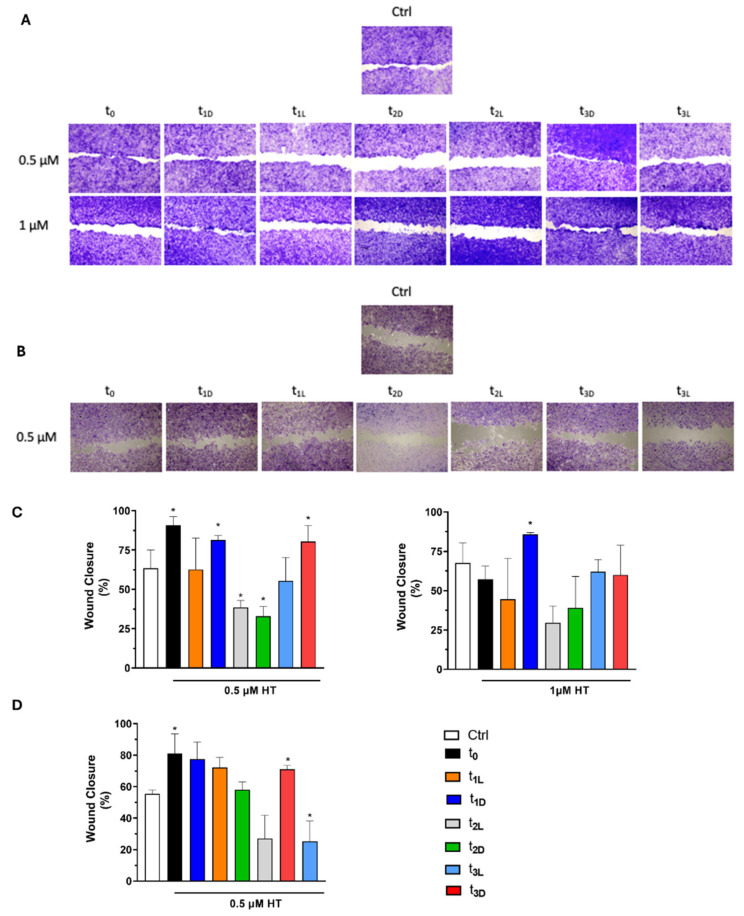
Representative images of the wound field observed at 18 h for untreated cells (Ctrl) and for cells treated with phenolic oil extracts at different concentrations: 0.5 and 1 µM for HaCaT (**A**) and 0.5 µM for HUVECs (**B**). Histogram plot together with ± SD of three independent experiments. * *p* < 0.05, treatments versus control (t test) for HaCaT (**C**) and HUVECs (**D**).

Cell migration and proliferation were assessed using a wound-healing assay to determine the ability of phenolic oil extracts to promote skin regeneration following injury. Using the same cell models (HaCat and HUVECs), and after making a scratch in the center of the seeded cells, phenolic EVOO extracts were administered at different concentrations: 0.5 and 1 µM in HT for HaCaT cells and 0.5 µM in HT for HUVECs. These concentrations were selected based on their lack of cytotoxicity evaluated by MTT assay. Then, the scratch was observed at an 18 h interval. Complete epithelization, defined as 100% wound closure in the shortest possible time, represents the most relevant clinical endpoint in wound management. An *in vitro* scratch test was used to mimic and evaluate the effects of phenolic oil extracts on the wound-healing process in HaCaT (Figure 6A) and HUVECs (Figure 6B) cells.

The migration of cells into the gap area was compared with the untreated control (Ctrl), which showed approximately 67.5% wound closure for HaCaT cells and 60.4% for HUVECs. In the keratinocyte cell model, the best results were obtained with 0.5 µM in HT of t_0_, t_1-D,_ and t_3-D_ extracts (Figure 6C) with a % of wound closure of 88.8%, 81.4%, and 80.7%, respectively. When 1 µM of HT was used, only t_1-D_ exhibited a positive effect, with 82.7% wound closure.

These data were further corroborated the HUVEC line, where t_0_, t_1-D,_ and t_1-L_ significantly enhanced wound closure to 71.3%, 77.5%, and 72.1%, respectively, compared to 60.4% in the Ctrl (Figure 6D). To summarize, t_0_ and t_1-D_ phenolic oil extracts in the lowest concentration tested, namely 0.5 µM in HT, demonstrated the highest efficacy in promoting wound closure in both cell lines. In fact, the scratch area closed significantly faster in the cells treated with the t_0_ and t_1-D_ extracts than in the Ctrl (Figure 6). The worst results were obtained with t_2-D_ and t_1L_ in both cell lines (Figure 6). The wound-healing effectiveness observed at time point t3 is likely due to the retention of certain beneficial compounds, such as *p*-coumaric acid, pinoresinol, and luteolin, despite a reduction in the overall phenolic content. These compounds are known to improve wound healing [15]. Additionally, the results of the t_1-L_ and t_2_ time points suggest that an optimal balance between the qualitative and quantitative characteristics of the phenolic extract is necessary to achieve beneficial effects. The reduced wound-healing efficacy observed in the t_1-L_, t_1-D_, t_2-L,_ and t_2-D_ samples might be explained by the degradation of oleacein and oleocanthal, two secoiridoid derivatives with well-documented anti-inflammatory, antioxidant, and tissue-regenerative properties [15]. These compounds are known to modulate key biological pathways involved in wound healing, such as the suppression of pro-inflammatory cytokines, the promotion of fibroblast proliferation, and the enhancement of collagen deposition. Their progressive degradation over time, likely due to oxidative or hydrolytic processes, could result in a diminished ability of the phenolic extract to support these critical cellular and molecular events. Consequently, the decline in their concentrations may compromise the overall bioactivity of the extract, leading to a reduced efficacy in promoting wound closure and tissue regeneration at later time points.

All these findings emphasize the ability of EVOO phenolic compounds to support skin health by promoting moisture retention and improving skin regeneration. Our study, in particular, shows that fresh phenolic extract (t_0_) and three months of storage in the dark (t_1-D_) are able to promote wound repair in the keratinocyte monolayer, an action that had already been demonstrated *in vitro* using phenolic extracts from olive oil rich in HT, Tyr, and oleocanthal, which induced an increase in fibronectin and α-actin expression and increased cell migration in the first hours after treatment, without changes in the cell cycle; results suggesting that phenolic compounds in oleocanthal-rich EVOO might contribute to wound healing through an action on fibroblasts related to tissue regeneration [42].

The efficacy of the t_0_ extract, which is rich in oleocanthal and oleacein, on the dermis supports previous *in vitro* research. Studies have shown that extra-virgin olive oil (EVOO) extracts can reduce the viability and migration of non-melanoma skin cancer cells, prevent colony and spheroid formation, and inhibit the proliferation of atypical keratinocytes stimulated by the epidermal growth factor [43].

What had already been demonstrated in the literature *in vitro* was that EVOOO extracts reduced non-melanoma skin cancer cell viability and migration, prevented colony and spheroid formation, and inhibited the proliferation of atypical keratinocytes stimulated with the epidermal growth factor [43].

Interestingly, all the solutions used in the assays were normalized based on HT levels, which remained relatively stable across the investigated samples (ranging from 5.72 to 13.34 mg/g of EVOO), compared to other phenols (Table 3). This result indicates that phenols other than HT are primarily responsible for the observed effects on skin moisture, wound-healing processes in keratinocytes and endothelial cells, and angiogenesis in HUVECs. Notably, oleacein and oleocanthal, present in high concentrations only at fresh time storage conditions (t_0_), emerge as key contributors. Already well-known for its anti-inflammatory and antioxidant properties, oleocanthal has been identified for its unique perceptual and anti-inflammatory characteristics, comparable to ibuprofen [44].

Recently, oleacein was also investigated by Cho and co-workers in primary normal human epidermal keratinocytes and fibroblasts, and it enhances proliferation and differentiation under low-calcium conditions [45]. In particular, oleacein upregulates key skin proteins like KRT10, IVL, FLG, and TGM1, essential for keratinocyte integrity, and improves cell adhesion, cytoskeletal reorganization, and intercellular communication.

Oleacein uniquely activates DNA repair (ATM/ATR-p53-MDM2 axis) to maintain differentiation under stress and enhances epithelial adhesion and keratinization, making it a promising agent for skin health.

A probably synergic effect of oleacein and oleocanthal in EVOO extract provides a strong combination for improving skin health by reinforcing the skin barrier, aiding wound healing, and regulating differentiation at the molecular level. Definitely, this synergistic interaction among various EVOO phenolics must be considered in future studies, as they complement each other in their anti-inflammatory and antioxidant effects [46]. Thus, oleacein and oleocanthal, alongside other EVOO phenolics, likely contribute to the overall health benefits associated with EVOO, including its potential use in topical applications.

The proper handling and preservation of EVOO phenolics are critical to maintaining their bioactive properties and maximizing their benefits in skincare products. In particular, two phenolic ingredients, namely oleacein and oleocanthal, must be considered of paramount importance to ensure their efficacy and safety in cosmetic formulations.

## 4. Conclusions

This study evaluated how real-world storage conditions affect Umbrian EVOO, simulating scenarios encountered during transportation, retail, and home storage. The findings reveal that improper storage significantly affects both quality parameters regulated by the European Commission Regulation and the aspects that contribute to EVOO’s health benefits and sensory properties. For this reason, the evolution of selected parameters was monitored in the “Rocca di Casalina” EVOO by storing the oil under light (L) exposure or in darkness (D) for three (t_1-L_ and t_1-D_), eight (t_2-L_ and t_2-D_), and twenty-four (t_3-L_ and t_3-D_) months. A marked deterioration in the peroxide value was observed after just three months (15.33 ± 0.04 and 14.98 ± 0.35 for t_1-L_ and t_1-D_, respectively). The phenolic fraction, particularly secoiridoids, lignans, and flavonoids, declined sharply within the first three months, followed by a gradual reduction over time. The loss of quality and nutritional values of the EVOO due to improper storage was also highlighted by the measurement of the total antioxidant capacity associated with the total phenol content. PCA revealed a clear separation of EVOO samples based on storage conditions, confirming their impact on phenolic composition and reinforcing the need for integrated analytical approaches to monitor EVOO quality and freshness. Additionally, color analysis emerged as a rapid and cost-effective quality indicator. Light exposure accelerated pigment degradation (carotenoids and chlorophyll), leading to significant color shifts, whereas dark storage helped preserve the oil’s original hue for a longer period.

The results of this study highlight the critical importance of proper EVOO storage, not only for maintaining its sensory and nutritional properties but also for preserving its therapeutic potential. Phenolic compounds, key bioactive molecules such as oleacein and oleocanthal, exhibit antioxidant and cell-regenerative effects, which are essential for wound healing and tissue repair. To preserve these bioactive compounds and maintain the oil’s health-promoting properties throughout its shelf life, appropriate storage practices are essential. These include using dark, airtight containers and maintaining controlled environmental conditions.

In conclusion, EVOO phenolic extracts represent a promising, eco-friendly solution for developing effective moisturizing and repairing skincare products, provided that their quality and stability are carefully managed throughout production and storage. Based on the obtained findings, future studies could focus on the development of specific formulations based on EVOO phenolics and explore the *in vivo* activity and synergistic effects with other natural bioactives, thereby opening exciting opportunities for the preparation of innovative nutraceutical products.

## Figures and Tables

**Table 1:** Colorimetric data of EVOO before storage (t_0_) and after three, eight, and twenty-four months of storage at room temperature in the light (t_1-L_, t_2-L,_ and t_3-L_) and in the dark (t_1-D_, t_2-D,_ and t_3-D_). Results are expressed as mean value ± standard deviation (*n* = 3).

Sample	L*	a*	b*	dE*ab	COLOR
t_0_	85.39 ± 0.00 ^a^	−11.23 ± 0.00 ^a^	72.1 ± 0.03 ^a^	1.78 ± 0.03 ^a^	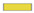
t_1-L_	87.44 ± 0.03 ^b^	−11.62 ± 0.02 ^b^	55.70 ± 0.04 ^b^	14.77 ± 0.04 ^b^	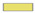
t_1-D_	86.80 ± 0.01 ^c^	−12.04 ± 0.01 ^c^	58.01 ± 0.00 ^c^	12.41 ± 0.00 ^c^	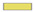
t_2-L_	87.31 ± 0.01 ^d^	−11.74 ± 0.01 ^b^	52.99 ± 0.03 ^d^	17.47 ± 0.03 ^d^	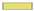
t_2-D_	86.28 ± 0.01 ^e^	−11.60 ± 0.01 ^d^	56.66 ± 0.04 ^e^	13.69 ± 0.04 ^e^	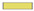
t_3-L_	86.79 ± 0.00 ^c^	−12.03 ± 0.02 ^c^	51.03 ± 0.02 ^f^	16.41 ± 0.00 ^f^	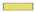
t_3-D_	86.81 ± 0.01 ^c^	−12.04 ± 0.01 ^c^	56.07 ± 0.02 ^g^	12.37 ± 0.01 ^c^	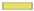

Different letters in each column indicate significant differences with *p*-value < 0.01. L* = black/white coordinate, a* = red/green color component, b* = yellow/blue coordinate, and dE*ab = overall color difference from the reference.

**Table 2:** Evolution of the free acidity and peroxide values before storage (t_0_) and after three, eight, and twenty-four months of storage at room temperature in the light (t_1-L_, t_2-L_, t_3-L_) and in the dark (t_1-D_, t_2-D_, t_3-D_).

EVOO Sample	Olive Oil Acidity (%)-Mean Value ^¤^	Peroxide Value (meq O_2_/kg)-Mean Value ^§^
t_0_	0.48 ± 0.01 ^a,^*	5.90 ± 0.13 ^a^
t_1-L_	0.38 ± 0.04 ^b,^*	15.33 ± 0.04 ^b^
t_1-D_	0.34 ± 0.00 ^b^	9.16 ± 0.20 ^c^
t_2-L_	0.35 ± 0.00 ^b^	14.98 ± 0.35 ^b,^*
t_2-D_	0.38 ± 0.04 ^b,^*	14.16 ± 0.11 ^d,^*
t_3-L_	0.87 ± 0.00 ^c^	17.69 ± 0.27 ^e^
t_3-D_	0.88 ± 0.02 ^c^	24.30 ± 0.52 ^f^

¤ extra-virgin olive oil is characterized by acidity < 0.8%. § the maximum peroxide value for extra-virgin olive oil is 20 meq O_2_/kg. Different superscript letters within the same column indicate significant differences with a *p*-value lower than 0.01. Asterisk (*) indicates significant differences with a *p*-value lower than 0.05.

**Table 3:** TPC and *in vitro* antioxidant activity determined on the polyphenol extracts from “Rocca di Casalina” EVOO before storage (t_0_) and after three, eight, and twenty-four months at room temperature in the light (t_1-L_, t_2-L_, t_3-L_) and in the dark (t_1-D_, t_2-D_, t_3-D_). Results are expressed as mean value ± standard deviation (*n* = 3).

EVOO Sample	TPC (mg GAE/kg)	DPPH (mg TE/kg)	ABTS (mg TE/kg)	FRAP (mg TE/kg)
t_0_	178.73 ± 4.07 ^a^	161.56 ± 5.38 ^a^	304.81 ± 3.72 ^a^	218.26 ± 1.58 ^a^
t_1-L_	121.90 ± 4.43 ^b^	116.51 ± 4.23 ^b^	327.56 ± 0.37 ^b^	178.78 ± 0.66 ^b^
t_1-D_	119.42 ± 2.37 ^b^	119.89 ± 1.85 ^b^	323.66 ± 2.41 ^b^	181.18 ± 4.26 ^b^
t_2-L_	140.33 ± 1.55 ^c^	142.73 ± 3.96 ^c^	324.64 ± 1.97 ^b^	198.84 ± 1.89 ^c^
t_2-D_	138.03 ± 4.63 ^c^	131.18 ± 1.01 ^b,c^	314.74 ± 5.75 ^a,b^	205.78 ± 1.64 ^a,c^
t_3-L_	63.19 ± 5.19 ^d^	93.91 ± 0.56 ^d^	172.52 ± 6.34 ^c^	133.04 ± 7.48 ^d^
t_3-D_	92.72 ± 3.18 ^e^	129.58 ± 5.48 ^b,c^	284.35 ± 3.99 ^c^	180.95 ± 3.24 ^b^

Different letters in each column indicate significant differences with a *p*-value < 0.01.

**Table 4:** The content of polyphenols in “Rocca di Casalina” EVOO was measured by HPLC-DAD, before storage (t_0_) and after three, eight, and twenty-four months of storage at room temperature in the light (t_1-L_, t_2-L_, t_3-L_) and in the dark (t_1-D_, t_2-D_, t_3-D_). Results are expressed as mean value ± standard deviation (n = 3).

EVOO Sample	Phenol Concentration (mg/g EVOO)					
	Hydroxytyrosol	Tyrosol	Vanillic Acid	*p*-Coumaric Acid	Veratric Acid	Oleacein
t_0_	5.72 ± 0.13 ^a^	2.96 ± 1.37 ^a^	0.55 ± 0.20 ^a^	0.10 ± 0.03 ^a^	0.04 ± 0.00	23.71 ± 0.69 ^a^
t_1-L_	7.07 ± 0.06 ^a,b^	2.31 ± 0.14 ^a^	0.41 ± 0.11 ^a,b^	0.05 ± 0.00 ^a,b^	nd	3.60 ± 0.64 ^b^
t_1-D_	8.89 ± 1.52 ^b,d^	3.12 ± 0.20 ^a^	0.14 ± 0.05 ^b,c^	0.10 ± 0.00 ^a^	nd	nd
t_2-L_	12.57 ± 0.38 ^c^	3.71 ± 0.12 ^b^	0.04 ± 0.02 ^c^	0.07 ± 0.00 ^a,b^	nd	0.62 ± 0.16 ^c^
t_2-D_	13.34 ± 0.33 ^c^	3.81 ± 0.54 ^a^	0.06 ± 0.02 ^b,c^	0.08 ± 0.00 ^a,b^	nd	nd
t_3-L_	8.19 ± 0.44 ^a,b^	2.65 ± 0.12 ^a^	0.07 ± 0.03 ^b,c^	0.03 ± 0.00 ^b^	nd	nd
t_3-D_	11.24 ± 0.52 ^c,d^	3.39 ± 0.14 ^a^	0.06 ± 0.00 ^b,c^	0.06 ± 0.00 ^b^	nd	nd
**EVOO sample**	**Phenol concentration (mg/g EVOO)**					
	**Oleuropein**	**Oleocanthal**	**Pinoresinol**	**Luteolin**	**Kaempferol**	**Apigenin**
t_0_	12.28 ± 0.56 ^a^	107.38 ± 1.13 ^a^	14.67 ± 0.20 ^a^	0.52 ± 0.02 ^a^	0.60 ± 0.04 ^a^	1.21 ± 0.08 ^a^
t_1-L_	9.04 ± 0.43 ^a,b^	42.10 ± 0.80 ^b^	6.19 ± 0.26 ^b^	0.25 ± 0.01 ^b^	nd	0.48 ± 0.00 ^b^
t_1-D_	5.80 ± 2.04 ^b,c^	23.22 ± 1.22 ^c^	5.51 ± 1.49 ^b^	0.25 ± 0.01 ^b^	0.12 ± 0.00 ^b^	0.57 ± 0.04 ^b^
t_2-L_	6.12 ± 0.13 ^b,c^	39.75 ± 3.52 ^b^	5.40 ± 0.33 ^b^	0.19 ± 0.01 ^b,c^	nd	0.37 ± 0.03 ^b,d^
t_2-D_	5.55 ± 0.12 ^c^	20.30 ± 1.93 ^c^	5.45 ± 0.30 ^b^	0.20 ± 0.04 ^b,c^	0.12 ± 0.02 ^b^	0.38 ± 0.14 ^b^
t_3-L_	4.48 ± 0.41 ^c^	21.31 ± 0.89 ^c^	4.97 ± 0.06 ^b^	0.17 ± 0.01 ^c^	0.01 ± 0.00 ^c^	0.04 ± 0.00 ^c^
t_3-D_	5.00 ± 0.30 ^c^	20.88 ± 1.31 ^c^	5.75 ± 0.15 ^b^	0.21 ± 0.01 ^b,c^	0.19 ± 0.01 ^d^	0.15 ± 0.01 ^c,d^

Different letters in each column indicate significant differences with a *p*-value < 0.01; nd = not detected.

## Data Availability

The original contributions presented in the study are included in the article/Supplementary Material, further inquiries can be directed to the corresponding authors.

## Supplementary Materials

The following supporting information can be downloaded at: https://www.mdpi.com/article/10.3390/foods14122104/s1, Figure S1: Trend of the free acidity (expressed as OA%) and peroxide value (PV, expressed as mEq O_2_/kg) of “Rocca di Casalina” EVOO samples, before and after storage at room temperature in the light (t_1-L_, t_2-L_, t_3-L_) and in the dark (t_1-D_, t_2-D_, t_3-D_). Figure S2: Correlation between (A) TPC vs. DPPH, ABTS and FRAP assays, (B) DPPH vs. ABTS, DPPH vs. FRAP, and FRAP vs. ABTS; (C) TPC vs. TAC total; (D) spectrophotometric assays (TPC and TAC) and total phenol concentration measured by HPLC-DAD. Table S1: Gas chromatographic analysis of the Rocca di Casalina olive oil fatty acid composition before storage (t_0_) and after eight and twenty-four months of storage at room temperature in the light (t_2-L_ and t_3-L_) and in the dark (t_2-D_ and t_3-D_). Values represent the mean of three determinations (n = 3) and three independent experiments. Table S2: Calibration data: regression equation, linearity range (μg/mL), coefficient of determination value (R^2^), and LOD and LOQ values. Tyrosol and luteolin were selected as representative standards, covering the explored range of polarity. Table S3: Method validation: evaluation of precision (RSD%) and accuracy (Recovery%) in the short- and long-term period (intra-day and inter-day) for tyrosol and lueteolin.

## Abbreviations

The following abbreviations are used in this manuscript:

ANGPTL2	Angiopoietin-like protein 2
3,4-DHPEA	3,4-dihydroxyphenyl-ethanol
DAD	Diode Array Detector
DMEM	Dulbecco’s modified Eagle’s medium
EVOO	extra-virgin olive oil
FBS	Fetal bovine serum
FGF2	Fibroblast growth factor 2
GAPDH	Glyceraldehyde 3-phosphate dehydrogenase
HA	hyaluronic acid
HAS	hyaluronan synthase
Hyal	hyaluronidase
HT	hydroxytyrosol
HUVECs	Human umbilical vein endothelial cells
IOOC	International Olive Oil Council
mEq	milliequivalent
OA %	oleic acid percentage
*p*-HPEA	*p*-hydroxyphenylethanol (Tyrosol)
PCA	principal component analysis
TAC	total antioxidant capacity
TGF-β	Transforming growth factor beta
Tyr	Tyrosol
TPC	total polyphenol content
VEGF	Vascular endothelial growth factor
VIP	Variable Importance in Projection

## References

[1] IOC (2024). International Olive Council COI/T.15/NC No 3/Rev. https://www.internationaloliveoil.org/wp-content/uploads/2024/11/TRADE-STANDARD-REV-20_EN.pdf.

[2] Serreli G., Deiana M. (2018). Biological relevance of extra virgin olive oil polyphenols metabolites. Antioxidants.

[3] Jimenez-Lopez C., Carpena M., Lourenco-Lopes C., Gallardo-Gomez M., Lorenzo J.M., Barba F.J., Prieto M.A., Simal-Gandara J. (2020). Bioactive compounds and quality of extra virgin olive oil. Foods.

[4] Piroddi M., Albini A., Fabiani R., Giovannelli L., Luceri C., Natella F., Rosignoli P., Rossi T., Taticchi A., Servili M. (2017). Nutrigenomics of extra-virgin olive oil: A review. Biofactors.

[5] Toledo E., Salas-Salvado J., Donat-Vargas C., Buil-Cosiales P., Estruch R., Ros E., Corella D., Fito M., Hu F.B., Aros F. (2015). Mediterranean diet and invasive breast cancer risk among women at high cardiovascular risk in the PREDIMED Trial: A Randomized Clinical Trial. JAMA Intern. Med..

[6] Guasch-Ferré M., Salas-Salvadó J., Ros E., Estruch R., Corella D., Fitó M., Martínez-González M.A. (2017). PREDIMED Investigators, The PREDIMED trial, Mediterranean diet and health outcomes: How strong is the evidence?. Nutr. Metab. Cardiovasc. Dis..

[7] Blasi F., Ianni F., Cossignani L. (2024). Phenolic profiling for geographical and varietal authentication of extra virgin olive oil. Trends Food Sci. Technol..

[8] Commission Delegated Regulation (EU) 2022/2104. (2022) Supplementing Regulation (EU) No. 1308/2013 of the European Parliament and of the Council as regards the Marketing Standards for Olive Oil and Which Repeals Regulation (EEC) No. 2568/91 of the Commission and the Implementing Regulation (EU) n. 29/2012 of the Commission. https://eur-lex.europa.eu/legal-content/IT/TXT/PDF/?uri=CELEX:32022R2104.

[9] Carrasco-Pancorbo A., Cerretani L., Bendini A., Segura-Carretero A., Gallina-Toschi T., Fernandez-Gutierrez A. (2005). Analytical determination of polyphenols in olive oils. J. Sep. Sci..

[10] Esposto S., Urbani S., Selvaggini R., Taticchi A., Gallina Toschi T., Daidone L., Bendini A., Veneziani G., Sordini B., Servili M. (2023). Potential of the oxidized form of the oleuropein aglycon to monitor the oil quality evolution of commercial extra-virgin olive oils. Foods.

[11] Castillo-Luna A., Criado-Navarro I., Ledesma-Escobar C.A., Lopez-Bascon M.A., Priego-Capote F. (2021). The decrease in the health benefits of extra virgin olive oil during storage is conditioned by the initial phenolic profile. Food Chem..

[12] Lazarus G.S., Cooper D.M., Knighton D.R., Margolis D.J., Pecoraro R.E., Rodeheaver G., Robson M.C. (1994). Definitions and guidelines for assessment of wounds and evaluation of healing. Arch. Dermatol. Res..

[13] Xue M., Jackson C.J. (2015). Extracellular matrix reorganization during wound healing and its impact on abnormal scarring. Adv. Wound Care.

[14] Zindle J.K., Wolinsky E., Bogie K.M. (2021). A review of animal models from 2015 to 2020 for preclinical chronic wounds relevant to human health. J. Tissue Viability.

[15] Melguizo-Rodriguez L., de Luna-Bertos E., Ramos-Torrecillas J., Illescas-Montesa R., Costela-Ruiz V.J., Garcia-Martinez O. (2021). Potential effects of phenolic compounds that can be found in olive oil on wound healing. Foods.

[16] Blasi F., Maurelli S., Cossignani L., D’Arco G., Simonetti M.S., Damiani P. (2009). Study of some experimental parameters in the synthesis of triacylglycerols with CLA isomers and structural analysis. J. Am. Oil Chem. Soc..

[17] Ianni F., Volpi C., Moretti S., Blasi F., Mondanelli G., Varfaj I., Galarini R., Sardella R., Di Renzo G.C., Cossignani L. (2022). In-depth characterization of phenolic profiling of Moraiolo extra-virgin olive oil extract and initial investigation of the inhibitory effect on Indoleamine-2,3-Dioxygenase (IDO1) enzyme. J. Pharm. Biomed. Anal..

[18] Mangiapelo L., Blasi F., Ianni F., Barola C., Galarini R., Abualzulof G.W., Sardella R., Volpi C., Cossignani L. (2023). Optimization of ultrasound-assisted extraction of chlorogenic acid from potato sprout waste and enhancement of the in vitro total antioxidant capacity. Antioxidants.

[19] Pagano C., Baiocchi C., Beccari T., Blasi F., Cossignani L., Ceccarini M.R., Orabona C., Orecchini E., Di Raimo E., Primavilla S. (2021). Emulgel loaded with flaxseed extracts as new therapeutic approach in wound treatment. Pharmaceutics.

[20] Ceccarini M.R., Ripanti F., Raggi V., Paciaroni A., Petrillo Comez C.L., Donato K., Bertelli M., Beccari T., Valentini L. (2023). Development of Salmon Sperm DNA/Regenerated Silk Bio-Based Films for Biomedical Studies on Human Keratinocyte HaCaT Cells under Solar Spectrum. J. Funct. Biomater..

[21] Dominici F., Imbriano A., Puglia D., Pagano C., Luzi F., Rafanelli A., Di Michele A., Bonacci F., Ceccarini M.R., Primavilla S. (2025). Starch-Based scaffold produced by FDM 3D printing technique as Innovative and biosustainable wound dressing. Eur. J. Pharm. Biopharm..

[22] Sikorska E., Caponio F., Bilancia M.T., Summo C., Pasqualone A., Khmelinskii I.V., Sikorski M. (2007). Changes in color of extra-virgin olive oil during storage. Pol. J. Food Nutr. Sci..

[23] Diez-Betriu A., Bustamante J., Romero A., Ninot A., Tres A., Vichi S., Guardiola F. (2023). Effect of the storage conditions and freezing speed on the color and chlorophyll profile of premium extra virgin olive oils. Foods.

[24] Gargouri B., Zribi A., Bouaziz M. (2015). Effect of containers on the quality of Chemlali olive oil during storage. J. Food Sci. Technol..

[25] Pristouri G., Badeka A., Kontominas M.G. (2010). Effect of packaging material headspace, oxygen and light transmission, temperature and storage time on quality characteristics of extra virgin olive oil. Food Control.

[26] Lazarou K., Tsagkaris A.S., Drakopoulou S., Kyriakopoulos A.M., Martakos I., Pentogenis M., Glyniadaki M., Kritikou E., Koupa A., Kostakis M. (2024). Long-term stability of extra virgin olive oil: Effects of filtration and refrigeration storage on the Kolovi variety. J. Sci. Food Agric..

[27] Sanmartin C., Venturi F., Sgherri C., Nari A., Macaluso M., Flamini G., Quartacci M.F., Taglieri I., Andrich G., Zinnai A. (2018). The effects of packaging and storage temperature on the shelf-life of extra virgin olive oil. Heliyon.

[28] Li X., Yang R., Lv C., Chen L., Zhang L., Ding X., Zhang W., Zhang Q., Hu C., Li P. (2018). Effect of Chlorophyll on Lipid Oxidation of Rapeseed Oil. Eur. J. Lipid Sci. Technol..

[29] Psomiadou E., Tsimidou M. (1998). Simultaneous HPLC Determination of Tocopherols, Carotenoids, and Chlorophylls for Monitoring Their Effect on Virgin Olive Oil Oxidation. J. Agric. Food Chem..

[30] Fan H., Xia S., Xiang J., Li Y., Ross M.O., Lim S.A., Yang F., Tu J., Xie L., Dougherty U. (2023). Trans-vaccenic acid reprograms CD8^+^ T cells and anti-tumour immunity. Nature.

[31] Pipoyan D., Stepanyan S., Stepanyan S., Beglaryan M., Costantini L., Molinari R., Merendino N. (2021). The effect of trans fatty acids on human health: Regulation and consumption patterns. Foods.

[32] Borges T.H., Pereira J.A., Cabrera–Vique C., Seiquer I. (2017). Study of the antioxidant potential of Arbequina extra virgin olive oils from Brazil and Spain applying combined models of simulated digestion and cell culture markers. J. Funct. Foods.

[33] Clodoveo M.L., Muraglia M., Crupi P., Hbaieb R., De Santis H.S., Desantis A., Corbo F. (2022). The tower of babel of pharma-food study on extra virgin olive oil polyphenols. Foods.

[34] Christodoulou M.C., Orellana Palacios J.C., Hesami G., Jafarzadeh S., Lorenzo J.M., Dominguez R., Moreno A., Hadidi M. (2022). Spectrophotometric methods for measurement of antioxidant activity in food and pharmaceuticals. Antioxidants.

[35] Geana E.I., Ciucure C.T., Apetrei I.M., Clodoveo M.L., Apetrei C. (2023). Discrimination of olive oil and extra-virgin olive oil from other vegetable oils by targeted and untargeted HRMS profiling of phenolic and triterpenic compounds combined with chemometrics. Int. J. Mol. Sci..

[36] Garcia B., Coelho J., Costa M., Pinto J., Paiva-Martins F. (2023). A simple method for the determination of bioactive antioxidants in virgin olive oils. J. Sci. Food Agric..

[37] Baccouri B., Sieren T., Rajhi I., Willenberg I. (2023). Characterization of the fingerprint profile of bioactive constituents of extra virgin olive oils from Peninsula Tunisian Cap Bon with regard to altitude. Eur. Food Res. Technol..

[38] Mousavi S., Mariotti R., Stanzione V., Pandolfi S., Mastio V., Baldoni L., Cultrera N.G.M. (2021). Evolution of extra virgin olive oil quality under different storage conditions. Foods.

[39] Castillo-Luna A., Ledesma-Escobar C.A., Gomez-Diaz R., Priego-Capote F. (2022). The secoiridoid profile of virgin olive oil conditions phenolic metabolism. Food Chem..

[40] Miho H., Moral J., Lopez-Gonzalez M.A., Diez C.M., Priego-Capote F. (2020). The phenolic profile of virgin olive oil is influenced by malaxation conditions and determines the oxidative stability. Food Chem..

[41] Frisina M., Bonacci S., Oliverio M., Nardi M., Vatrano T.P., Procopio A. (2023). Storage Effects on bioactive phenols in Calabrian monovarietal extra virgin olive oils based on the EFSA health claim. Foods.

[42] Gonzalez-Acedo A., Ramos-Torrecillas J., Illescas-Montes R., Costela-Ruiz V.J., Ruiz C., Melguizo-Rodriguez L., Garcia-Martinez O. (2023). The benefits of olive oil for skin health: Study on the effect of hydroxytyrosol, tyrosol, and oleocanthal on human fibroblasts. Nutrients.

[43] Polini B., Digiacomo M., Carpi S., Bertini S., Gado F., Saccomanni G., Macchia M., Nieri P., Manera C., Fogli S. (2018). Oleocanthal and oleacein contribute to the in vitro therapeutic potential of extra virgin oil-derived extracts in non-melanoma skin cancer. Toxicol. Vitr..

[44] Francisco V., Ruiz-Fernandez C., Lahera V., Lago F., Pino J., Skaltsounis L., Gonzalez-Gay M.A., Mobasheri A., Gomez R., Scotece M. (2019). Natural molecules for healthy lifestyles: Oleocanthal from extra virgin olive oil. J. Agric. Food Chem..

[45] Cho J., Bejaoui M., Isoda H. (2025). Regulation of keratinocyte proliferation and differentiation by secoiridoid oleacein in monoculture and fibroblast co-culture models. Biomed. Pharmacother..

[46] Parkinson L., Keast R. (2014). Oleocanthal, a phenolic derived from virgin olive oil: A review of the beneficial effects on inflammatory disease. Int. J. Mol. Sci..

