# Supplementary material for: Impact of Extra-Virgin Olive Oil Storage Conditions on Phenolic Content and Wound-Healing Properties
Source: Foods. 2025 Jun 15;14(12):2104. doi: 10.3390/foods14122104 (PMC12191701; doi:10.3390/foods14122104)
Supplement: Supplementary file 1 [file foods-14-02104-s001.zip › foods-3674147-supplementary.pdf]

foods

MDPI

Supplementary Material

Impact of extra-virgin olive oil storage conditions on phenolic content and wound healing properties

Francesca Blasi, [1,†] **Maria Rachele Ceccarini,** [2,†] **Stefano Bistarelli,** [3] **Francesco Galli,** [2] **Lina Cossignani,** [1] **Desirée Bartolini,** [2,*] **and Federica Ianni** [1,*]

[1] Section of Food, Biochemical, Physiological and Nutritional Sciences, Department of Pharmaceutical Sciences, University of Perugia, 06126 Perugia, Italy; francesca.blasi@unipg.it (F.B.); lina.cossignani@unipg.it (L.C.)

[2] Section of Morphological Biomolecular Nutraceutical and Health Sciences, Department of Pharmaceutical Sciences, University of Perugia, 06126 Perugia, Italy; mariarachele.ceccarini@unipg.it (M.C.); francesco.galli@unipg.it (F.G.)

[3] Department of Mathematics and Computer Science, University of Perugia, 06123 Perugia, Italy; stefano.bistarelli@unipg.it (S.B.)

* Correspondence: desiree.bartolini@unipg.it (D.B.) Tel.: +39-075-5857445; federica.ianni@unipg.it (F.I.) Tel.: +39-075-5857955

† These authors contributed equally to this work

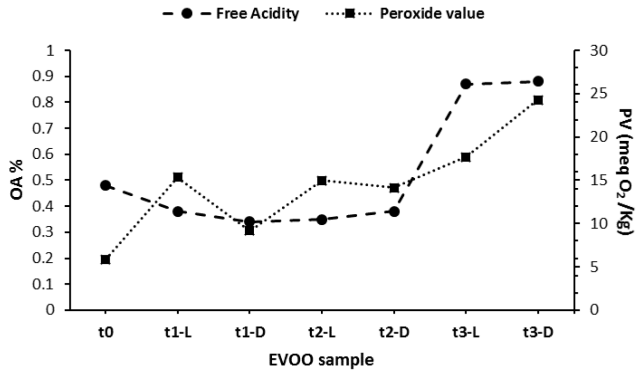

Figure S1. Trend of the free acidity (expressed as OA%) and peroxide value (PV, expressed as mEq O_2/kg) of "Rocca di Casalina" EVOO samples, before and after storage at room temperature in the light ($t_{1\text{-L}}$, $t_{2\text{-L}}$, $t_{3\text{-L}}$) and in the dark ($t_{1\text{-D}}$, $t_{2\text{-D}}$, $t_{3\text{-D}}$).

Table S1. Gas chromatographic analysis of the Rocca di Casalina olive oil fatty acid composition before storage (t_0) and after eight and twenty-four months of storage at room temperature in the light ($t_{2\text{-}L}$ and $t_{3\text{-}L}$) and in the dark ($t_{2\text{-}D}$ and $t_{3\text{-}D}$). Values represent the mean of three determinations (*n*=3) and three independent experiments.

Fatty acid	t_0	$t_{1\text{-}L}$	$t_{1\text{-}D}$	$t_{2\text{-}L}$	$t_{2\text{-}D}$	$t_{3\text{-}L}$	$t_{3\text{-}D}$
C16:0	15.44±0.32	14.24±0.43	14.53±0.35	14.86±0.05	15.00±0.00	14.33±0.71	14.70±0.30
C16:1 n9	0.11±0.01	0.08±0.01	0.06±0.01	0.10±0.01	0.13±0.00	0.07±0.06	0.09±0.00
C16:1 n7	1.13±0.02	1.06±0.07	1.04±0.01	1.09±0.02	1.14±0.03	1.11±0.13	1.14±0.18
C17:0	0.10±0.00	0.08±0.00	0.09±0.01	0.10±0.01	0.11±0.04	0.17±0.09	0.08±0.03
C18:0	1.90±0.02	2.11±0.07	1.97±0.11	1.97±0.01	1.94±0.00	1.82±0.12	1.78±0.14
C18:1 n9	69.53±0.17	70.26±0.03	70.57±0.48	69.89±0.25	69.68±0.04	70.14±1.12	70.13±0.47
C18:1 n7	2.78±0.05	3.58±0.30	2.96±0.07	2.79±0.18	2.95±0.02	3.75±0.52	4.22±0.51
C18:2 n6	7.73±0.06	7.63±0.20	7.82±0.12	7.88±0.04	7.77±0.00	7.78±0.01	7.09±0.43
C18:3 n3	0.29±0.02	0.24±0.04	0.21±0.01	0.28±0.02	0.28±0.02	nd	nd
C20:0	0.64±0.00	0.44±0.09	0.41±0.01	0.69±0.03	0.66±0.02	0.57±0.02	0.43±0.19
C20:1	0.27±0.03	0.17±0.01	0.21±0.10	0.27±0.01	0.27±0.01	0.16±0.01	0.35±0.23
C22:0	0.06±0.00	0.13±0.04	0.12±0.03	0.08±0.01	0.08±0.00	0.08±0.00	0.03±0.00

Table S2. Calibration data: regression equation, linearity range (μg/mL), coefficient of determination value (R^2), LOD and LOQ values. Tyrosol and luteolin were selected as representative standards, covering the explored range of polarity.

Analyte	Regression Equation	Linearity range (μg/mL)	R^2	LOD* (μg/mL)	LOQ* (μg/mL)
**Tyrosol**	y = 14337.68(±150.42)x + 8923.50(±1552.77)	1.25-20	0.9997	0.36	1.08
**Luteolin**	y = 63779.47(±658.79)x + 51698.88(±6796.38)	1.25-20	0.9997	0.35	1.07

*LOD and LOQ values were calculated as the standard deviation of the response (σ_y) on the slope of the calibration curve (b), according to the equations: $C_{LOD}= 3.3(\sigma_y/b)$ and $C_{LOQ} = 10(\sigma_y/b)$.

Table S3. Method validation: evaluation of precision (RSD%) and accuracy (Recovery%) in the short and long-term period (intra-day and inter-day) for tyrosol and lueteolin.

Theroetical Conc. (µg/mL)	Intra-day mean concentration (µg/mL)			Intra-day Precision (RSD%)			Intra-day Accuracy (Recovery)			Inter-day mean Precision (RSD%)	Inter-day mean Accuracy (Recovery)
	Day1	Day2	Day3	Day1	Day2	Day3	Day1	Day2	Day3		
Tyrosol 2.45	2.45	2.58	2.46	0.05	0.09	0.07	99.48	104.61	100.43	0.09	99.92
Tyrosol 0.25	0.52	0.50	0.51	0.01	0.01	0.02	103.72	100.35	102.86	0.01	102.31
Luteolin 2.45	2.47	2.54	2.51	0.10	0.08	0.06	100.15	102.81	102.43	0.08	100.22
Luteolin 0.20	0.25	0.25	0.26	0.00	0.00	0.00	100.14	98.83	102.40	0.00	100.46

Intra-day and Inter-day evaluation: analysis of 3 replicates of each external set within one day and for three consecutive days.

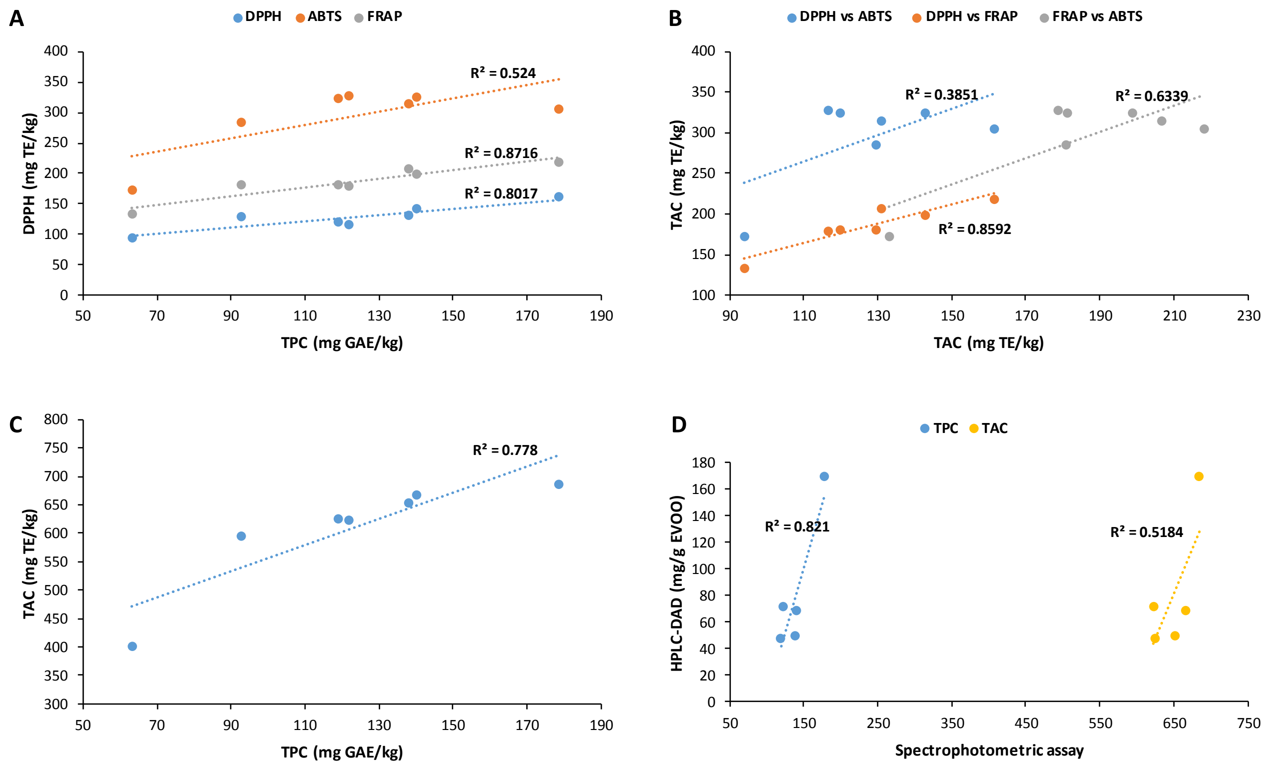

Figure S2. Correlation between A) TPC *vs* DPPH, ABTS and FRAP assays, B) DPPH *vs* ABTS, DPPH *vs* FRAP, FRAP *vs* ABTS; C) TPC *vs* TAC total; D) spectrophotometric assays (TPC and TAC) and total phenol concentration measured by HPLC-DAD.
